# Aminoadipate-semialdehyde synthase, a potential target for substrate reduction therapy in glutaric aciduria type 1

**DOI:** 10.1038/s41598-026-44377-9

**Published:** 2026-03-31

**Authors:** Celine Saad, Sabine Jung-Klawitter, Bianca Dimitrov, Juan Antonio Aguilar-Pimentel, Lore Becker, Patricia da Silva-Buttkus, Nathalia R. V. Dragano, Lillian Garrett, Sabine M. Hölter, Birgit Rathkolb, Adrián Sanz-Moreno, Nadine Spielmann, Helmut Fuchs, Valerie Gailus-Durner, Christian P. Schaaf, Giancarlo la Marca, Roberta Damiano, Dirk J. Lefeber, Udo Engelke, the CHARLIE Consortium, Dirk J. Lefeber, Clara C. D. M. van Karnebeek, Alex Garanto Iglesias, Carole Linster, Curtis R. Coughlin, Blair R. Leavitt, Christina Fillat, Martin Hrabe de Angelis, Sander M. Houten, Stefan Kölker

**Affiliations:** 1 Medical Faculty of Heidelberg, Department of Pediatrics I, Division of Pediatric Neurology and Metabolic Medicine, Heidelberg University, 69120 Heidelberg, Germany; 2 Institute of Experimental Genetics and German Mouse Clinic, Helmholtz Zentrum München, German Research Center for Environmental Health, 85764 Neuherberg, Germany; 3 German Center for Diabetes Research (DZD), 85764 Neuherberg, Germany; 4 Institute of Molecular Animal Breeding and Biotechnology, Gene Center, Ludwig- Maximilians-Universität München, 81377 Munich, Germany; 5 Chair of Experimental Genetics, TUM School of Life Sciences, Technische Universität München, 85354 Freising, Germany; 6 Institute of Human Genetics, Heidelberg University, 69120 Heidelberg, Germany; 7 Newborn Screening Clinical Biochemistry and Clinical Pharmacy Laboratory, Department of Neuroscience and Medical Genetics Meyer Children’s Hospital IRCCS, Florence, 50139 Italy; 8 Department of Experimental and Clinical Biomedical Sciences, University of Florence, Florence, Italy; 9 Translational Metabolic Laboratory, Department of Human Genetics, Radboud University Medical Center, Nijmegen, Netherlands; 10 Department of Neurology, Donders Institute for Brain, Cognition and Behavior, Radboud University Medical Center, Nijmegen, Netherlands; 11 Department of Genetics and Genomic Sciences, Icahn School of Medicine at Mount Sinai, New York, NY 10029 USA; 12 Department of Pediatrics and Human Genetics, Emma Center for Personalized Medicine, Amsterdam University Medical Centers, Amsterdam, The Netherlands; 13 United for Metabolic Diseases, Amsterdam, The Netherlands; 14 Amsterdam Gastroenterology Endocrinology Metabolism, Amsterdam University Medical Centers, Amsterdam, The Netherlands; 15 Department of Pediatrics, Radboud University Medical Center, Nijmegen, The Netherlands; 16 Radboud Institute for Molecular Life Sciences, Radboud University Medical Center, Nijmegen, The Netherlands; 17 Amalia Childrens Hospital, Radboud University Medical Center, Nijmegen, The Netherlands; 18 Department of Human Genetics, Radboud University Medical Center, Nijmegen, The Netherlands; 19 Luxembourg Centre for Systems Biomedicine, University of Luxembourg, L-4362 Esch-Belval, Luxembourg; 20 Section of Clinical Genetics and Metabolism, Department of Pediatrics, University of Colorado Anschutz Medical Campus, Aurora, CO USA; 21 Department of Medical Genetics, Faculty of Medicine, University of British Columbia, 317-2194 Health Sciences Mall, Vancouver, BC V6T 1Z3 Canada; 22 Centre for Molecular Medicine and Therapeutics, BC Childrens Hospital Research Institute, 938 West 28th Avenue, Vancouver, BC V5Z 4H4 Canada; 23 Institut d’Investigacions Biomèdiques August Pi i Sunyer (IDIBAPS), Barcelona, Spain; 24 Facultat de Medicina i Ciències de la Salut, Universitat de Barcelona (UB), Barcelona, Spain; 25 Centro de Investigación Biomédica en Red de Enfermedades Raras (CIBERER), Barcelona, Spain

**Keywords:** Glutaric aciduria type 1, Glutaric acid, Aminoadipate-semialdehyde synthetase, Glutaryl-CoA dehydrogenase, Biochemistry, Diseases, Drug discovery, Medical research, Neurology, Neuroscience

## Abstract

Glutaric aciduria type 1 is caused by inherited deficiency of glutaryl-CoA dehydrogenase and subsequent accumulation of neurotoxic metabolites. Clinically, the disease is characterized by striatal damage and dystonic movement disorder in untreated infants. Despite newborn screening and pre-symptomatic therapy start, about one-third of patients still develop neurological symptoms. Furthermore, progressive white matter changes and chronic kidney disease highlights the need for improved therapies. To elucidate the potential of substrate reduction therapy for GA1 we investigated whether aminoadipate-semialdehyde synthetase, the first enzyme of the lysine oxidation pathway, could serve as therapeutic target. Therefore, we studied whether *Gcdh* knockout (KO) mice, a known animal model for GA1, were rescued by additional knockout of *Aass*. *Gcdh/Aass* KO mice were clinically indistinguishable from wild-type mice and showed a marked reduction of glutaric acid in brain (20.9 µg/mg protein vs. 59.2 µg/mg protein; *p* = 0.001), liver (23.5 µg/mg protein vs. 104.8 µg/mg protein; *p* = 0.001), and urine (11.9 mol/mol creatinine vs. 166.5 mol/mol creatinine; *p* = 0.001). The effect was less pronounced for 3-hydroxyglutaric acid. Unlike *Gcdh* KO mice, *Gcdh/Aass* KO mice did not develop a severe phenotype under high-lysine diet. In conclusion, knockout of *Aass* partially rescues the severe phenotype of *Gcdh* KO mice, providing a potential therapeutic target.

**Supplementary Information:**

The online version contains supplementary material available at 10.1038/s41598-026-44377-9.

## Introduction

**Fig. 1:**
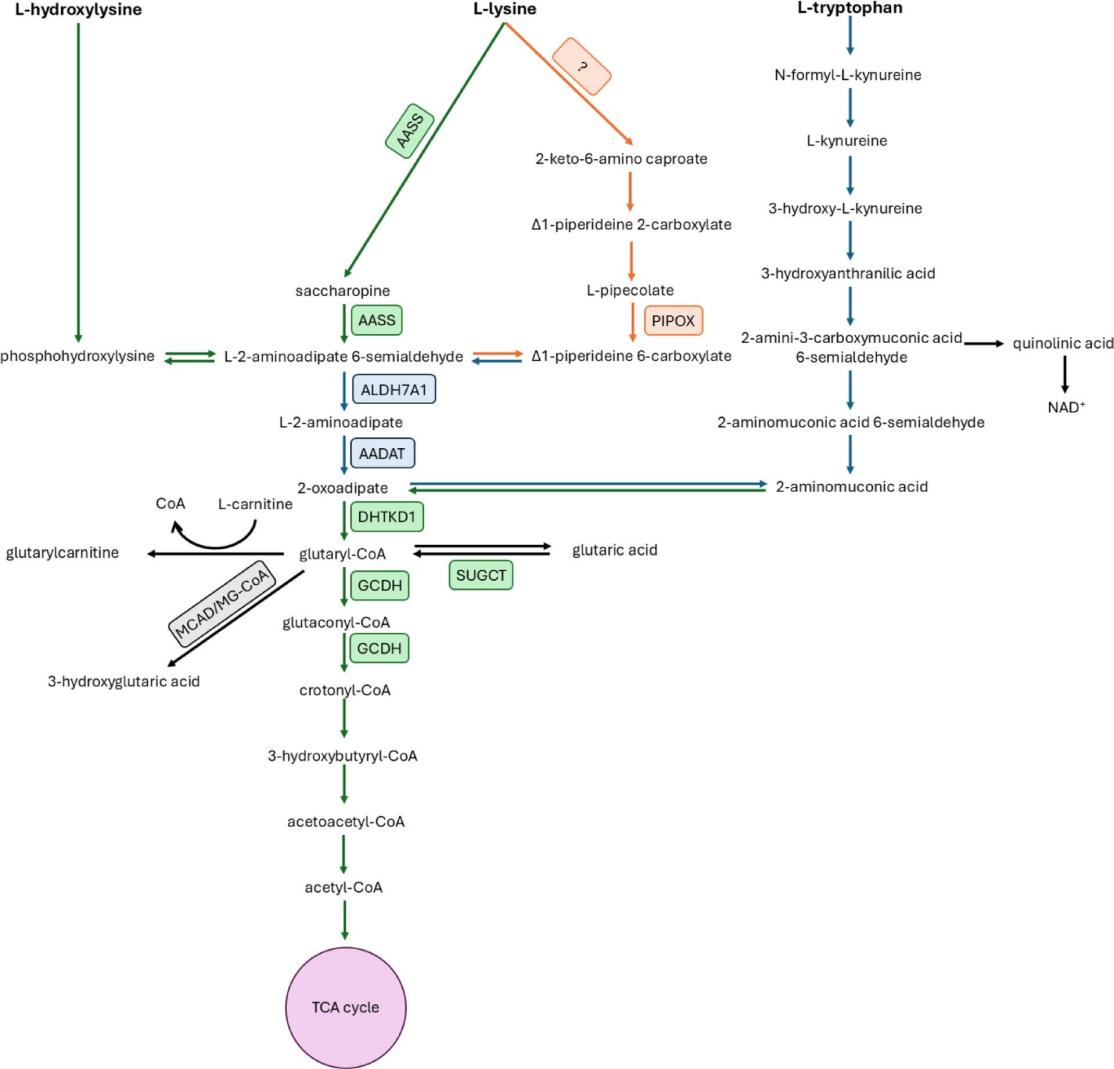
Degradation pathways of L-lysine, L-hydroxylysine and L-tryptophan. The deficiency of glutaryl-CoA dehydrogenase (GCDH) results in the accumulation and urinary excretion of the metabolites glutaric acid (GA), 3-hydroxyglutaric acid (3-OH-GA), and glutarylcarnitine (C5DC). Abbreviations used: AASS, aminoadipic acid semialdehyde synthase; ALDH7A1, Aldehyde dehydrogenase 7; AADAT, 2 aminoadipate aminotransferase; DHTKD1, dehydrogenase E1 and transketolase domain containing protein 1; MCAD, medium-chain acyl-CoA dehydrogenase; TCA cycle, tricarboxylic acid cycle. Enzymes are represented by squares. Reactions catalyzed in the mitochondria are labeled in green, cytoplasmic reactions are labeled in blue and peroxisomal reactions are labeled in orange.

Glutaric aciduria type 1 (GA1; MIM #231670) is a rare neurometabolic disorder caused by biallelic pathogenic variants in the *GCDH* gene, localized on chromosome 19p13.13, which results in deficiency of glutaryl-CoA dehydrogenase (GCDH; EC 1.3.8.6), a mitochondrial flavin adenine dinucleotide (FAD)-dependent enzyme. This enzyme is involved in the degradation of L-lysine, L-hydroxylysine, and L-tryptophan (Fig. 1). Biochemically, GCDH deficiency leads to the accumulation of glutaryl-CoA, glutaric acid (GA), 3-hydroxyglutaric acid (3OHGA), and glutaryl-carnitine (Fig. 1). GA is utilized for metabolic work-up and patient identification in newborn screening. Loss of GCDH activity is associated with a strong accumulation of these metabolites (high excreter (HE) phenotype), while a residual activity of 3–30% leads to a low excreter (LE) phenotype, often with intermittently normal metabolic test results in blood and urine. GA1 has an estimated prevalence of approximately 1 in 100,000 newborns worldwide. However, high-risk populations with a prevalence of up to 1 in 300 newborns are known^1,2^.

The underlying pathological mechanisms are still subject to debate and have been summarized in the overlapping toxic metabolite, entrapment, and CASTOR (CoA sequestration, toxicity and redistribution) hypotheses^3^. These hypotheses are based on the finding that in some inherited metabolic diseases accumulating metabolites exceed a threshold for the induction of adverse reactions, such as inhibition of the 2-oxoglutarate dehydrogenase complex by glutaryl-CoA in GA1^4,5^. Accumulation of such toxic metabolites is facilitated by their limited ability to cross biological barriers, such as the inner mitochondrial membrane (glutaryl-CoA) or the blood brain barrier (GA, 3OHGA^6,7^. Within mitochondria, metabolite entrapment may have a negative impact on the free CoA pool through CoA sequestration^8^. Furthermore, intramitochondrial accumulation of acyl-CoA may result in chronically enhanced nonenzymatic, metabolite-sensitive protein acylation and thus chronically changed enzymatic activity^9^. In line with this, *Gcdh* KO mice, an animal model for GA1, show increased glutarylation of mitochondrial proteins with an unexpected focus on astrocytes^10^.

GA1 is considered a treatable condition. However, therapeutic efficacy critically depends on the age at which treatment is initiated and the quality of treatment and care. Newborn screening (NBS) has been shown to be the prerequisite of a beneficial neurological outcome for individuals with GA1, while treatment, that starts after the manifestation of irreversible striatal damage with subsequent dystonic movement disorder, is ineffective^11–13^. Evidence-based recommendations for diagnosing and managing individuals with GA1 have been revised recently^14^. Metabolic treatment includes low-lysine diet preferably with lysine-free, tryptophan-reduced and arginine-fortified amino acid supplements, and carnitine supplementation. Emergency treatment is available with enhanced caloric intake and transient stop of protein intake during catabolic stress^15,16^. This combined treatment aims at reducing the accumulation of neurotoxic dicarboxylic compounds through limiting the uptake and degradation of L-lysine, the quantitatively major amino acid precursor^5^. Although this treatment is regarded safe and effective, approximately one third of early treated patients still develop substantial neurological symptoms^17^, often as a consequence of limited adherence to dietary recommendations^18^. Furthermore, currently available therapy does not seem to impact chronic kidney disease and progressive white matter change^12,19^, and inadequate dietary management increases the risk of malnutrition^20^. This highlights the need for safer and more effective therapies for individuals with GA1, that reliably protect against neurological and non-neurological disease manifestation throughout lifetime. In addition to the recently published approaches of gene replacement therapy for GA1^21^, substrate reduction therapy seems an excellent therapeutic option. The latter has been successfully implemented for other inherited metabolic diseases, such as nitisinone treatment in tyrosinemia type 1 as well as eliglustat and miglustat for Gaucher disease^22,23^. Similar approaches may be beneficial for GA1.

It has been reported^24^ that *Gcdh* KO mice, which resemble the biochemical phenotype of the HE group of GA1 patients, do not spontaneously develop a severe neurological phenotype under standard diet, while exposure to high-lysine diet (HLD) induces a severe phenotype with motor deficits, seizures, and encephalopathy finally leading to death in the majority of exposed *Gcdh* KO mice. First attempts to establish a pharmacological substrate reduction therapy (SRT) in this animal model using DHTKD1 as a target failed. Dehydrogenase E1 and transketolase domain‑containing protein 1 (DHTKD1) is a mitochondrial enzyme involved in lysine and tryptophan catabolism (Fig. 1). The genetic DHTKD1 inhibition did not rescue the biochemical and clinical phenotype of *Gcdh* KO mice. The reason for this is that 2-oxoglutarate dehydrogenase complex is not specific for 2-oxoglutarate as a substrate but can also use, although with low affinity, 2-oxoadipate, thereby circumventing the induced block of the DHTKD1-containing 2-oxoadipate dehydrogenase complex^25,26^.

Aminoadipate-semialdehyde synthase (AASS) is a mitochondrial enzyme that catalyzes the first two steps of the saccharopine pathway (Fig. 1), the major lysine degradation route in mammals. AASS is a bifunctional enzyme comprising two domains. First, a lysine-oxoglutarate reductase (LOR), which converts lysine to saccharopine, and second, a saccharopine dehydrogenase (SDH) domain, converting saccharopine to aminoadipate-semialdehyde. Through these steps, AASS provides substrates that are further metabolized to glutaryl-CoA along this pathway. Although it has been controversially discussed, whether and under which circumstances AASS is expressed in the brain^5^, a recent publication indicates a functional expression of AASS in mouse brain^27^.

Deficiency of AASS has been associated with benign clinical phenotypes, i.e., hyperlysinemia type I and II, which lead to accumulation of lysine in plasma and urine^28,29^. Hyperlysinemia type I is caused by a complete loss of LOR and SDH activities, while type II is induced by selective loss of SDH activity, leading to saccharopine accumulation. Hyperlysinemia is characterized by elevated plasma lysine concentrations with no underlying pathological traits. The benign phenotype of inherited AASS deficiency^30^ suggests, that pharmacological inhibition of AASS might be a safe and effective therapeutic strategy for individuals with GA1.

The aim of this study was to elucidate the biochemical and clinical impact of AASS knockout in *Gcdh* KO mice, thereby validating a new druggable target for substrate reduction therapy in GA1.

## Results

### Characterization of *Gcdh* KO and *Gcdh/Aass* KO mice under standard diet

**Fig. 2:**
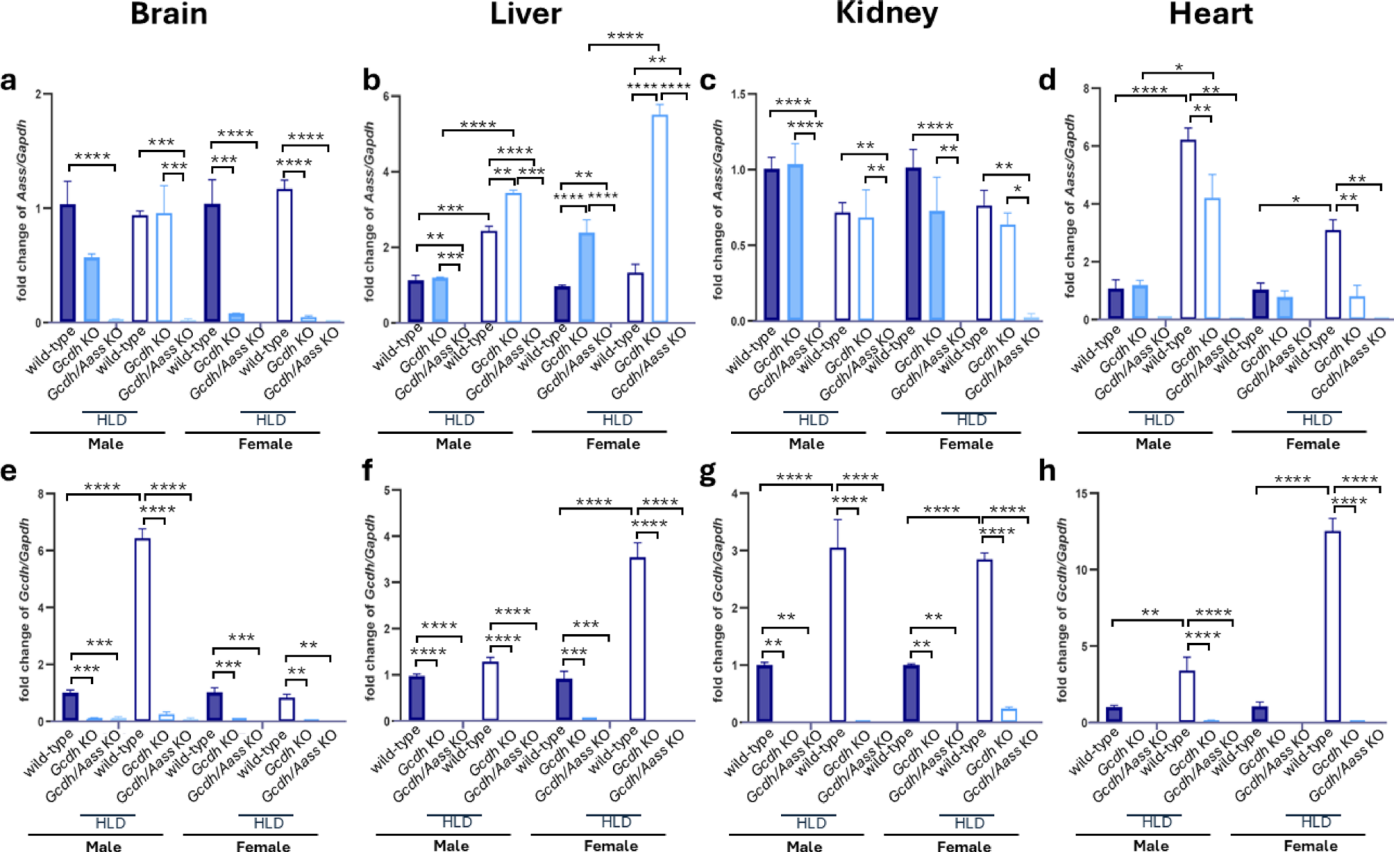
Molecular characterization of mice at 4 weeks of age under standard diet or HLD. Expression of *Aass* (**a**-**d**) and *Gcdh* (**e**-**h**) mRNA in brain (a, e), liver (b, f), kidney (c, g), and heart (d, h) of male and female WT, *Gcdh KO*, and *Gcdh/Aass KO* mice with or without HLD and normalized to *Gapdh* with healthy controls set as 1. Statistical analysis was performed using one-way ANOVA followed by Bonferroni’s multiple comparisons test. Significance * p value < 0.05, ** for *p* ≤ 0.01, *** for *p* ≤ 0.001; *n* = 3 for all groups. Filled bar: standard diet. Empty bar: HLD. Abbreviations: *Aass*, aminoadipate-semialdehyde synthase; *Gcdh*, glutaryl-CoA dehydrogenase; HLD, high lysine diet; KO, knockout.

**Fig. 3:**
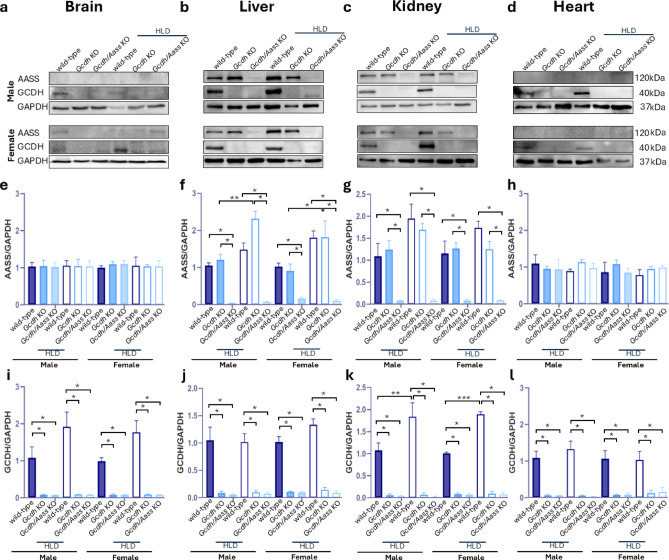
Protein expression in mice at 4 weeks of age under standard diet or HLD. (a-d) AASS and GCDH protein expression in brain (**a**), liver (**b**), kidney (**c**), and heart (**d**) of male and female mice with or without HLD. GAPDH served as loading control and for quantification of AASS (**e**-**h**) and GCDH (**j**-**l**) expression. Statistical analysis was performed using one-way ANOVA followed by Tukey’s multiple comparisons test. Significance * p value < 0.05, ** for *p* ≤ 0.01, *** for *p* ≤ 0.001. *n* = 3 for all groups. Filled bar, standard diet: Empty bar, HLD. Abbreviations: *Aass*, aminoadipate-semialdehyde synthase; *Gcdh*, glutaryl-CoA dehydrogenase; HLD, high lysine diet; kDA, kilodalton; KO, knockout.

In addition to the previously characterized *Gcdh* KO mice^24^, we established *Gcdh/Aass* KO mice, aiming to reduce the generation of neurotoxic metabolites through blocking the first step of the saccharopine branch of the lysine oxidation pathway. To test this hypothesis and to check the validity of these mouse models, we first studied the expression of *Gcdh* and *Aass*. As expected, *Gcdh* was not expressed in the analyzed organs of *Gcdh* KO mice (Fig. 2e-h), and *Gcdh* (Fig. 2e-h) and *Aass* (Fig. 2a-d) transcripts were not expressed in *Gcdh/Aass* KO mice. In analogy, the GCDH protein was not expressed in organs of *Gcdh* KO mice (Fig. 3a-d, i-l), and GCDH and AASS were not expressed in *Gcdh/Aass* KO mice (Fig. 3a-l).

**Fig. 4:**
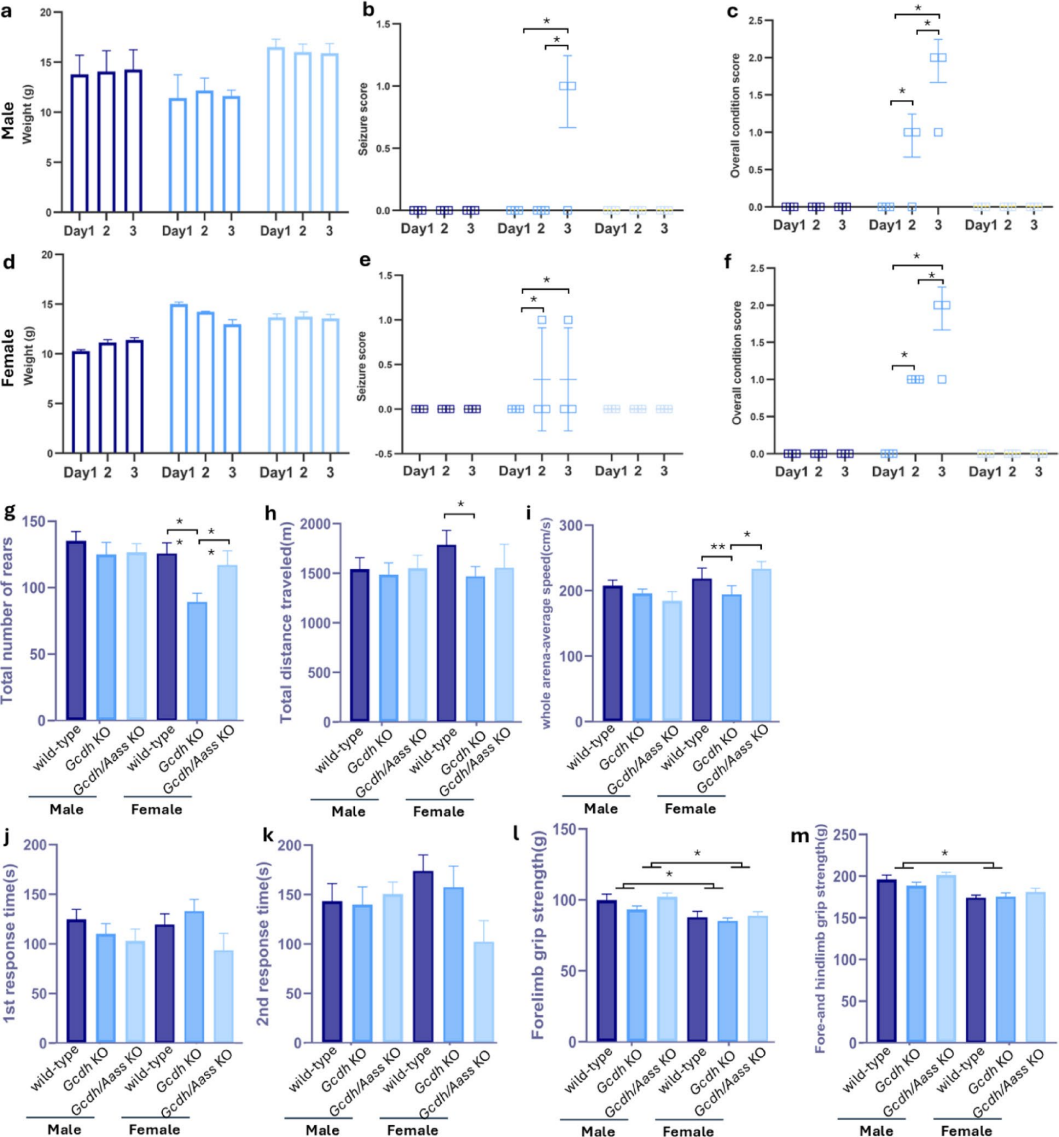
Mouse score sheet in 4-week-old mice shows behavioral rescue in *Gcdh/Aass* KO mice. Weight change during HLD in males (**a**) and females (**d**), seizure-like behavior during HLD in males (**b**) and females (**e**), and overall condition including the percentage of weight loss, seizure like behavior, and the decrease in movement in males (**c**) and females (**f**). *n* = 3 for all groups. Open field test result measurements in 9-week-old mice under standard diet include (**g**) total number of rears, (**H**) total distance traveled, and (**i**) average speed. Grip strength test in 10-week-old mice includes (**j**) forelimb grip strength, (**n**) forelimb and hindlimb grip strength. Hotplate test in 11-week-old mice includes (**k**) time for first response, (**l**) time for second response, and (**m**) percentage of first reaction either shake or lick of the limb. *n* ≥ 10–15 for all groups. Statistical analysis was performed using one-way ANOVA followed by Tukey’s multiple comparisons test. Significance * p value < 0.05, ** for *p* ≤ 0.01. Filled bar, standard diet; empty bar, HLD. Abbreviations: *Aass*, aminoadipate-semialdehyde synthase; *Gcdh*, glutaryl-CoA dehydrogenase; HLD, high lysine diet; KO, knockout.

Since in many neurological diseases, males and females can have different susceptibilities due to hormonal influences, metabolic rate variations, and differences in mitochondrial function^31,32^, we wondered whether we could identify sex-specific differences in the studied mouse models. Transcript and protein levels revealed no differences between the sexes (Figs. 2 and 3). Furthermore, male and female GA1 mice presented with a similar phenotype at 4 weeks of age, even under HLD (Fig. 4a-f). However, 9-week-old female *Gcdh* KO mice showed a more pronounced phenotype compared to WT animals even without HLD (Fig. 4g-m). Specifically, they presented with a decrease in rearing behavior, distance travelled, and speed (Fig. 4g-i).

### Comparison of the biochemical and neurobehavioral phenotype of *Gcdh* KO and *Gcdh/Aass* KO under standard diet

**Fig. 5:**
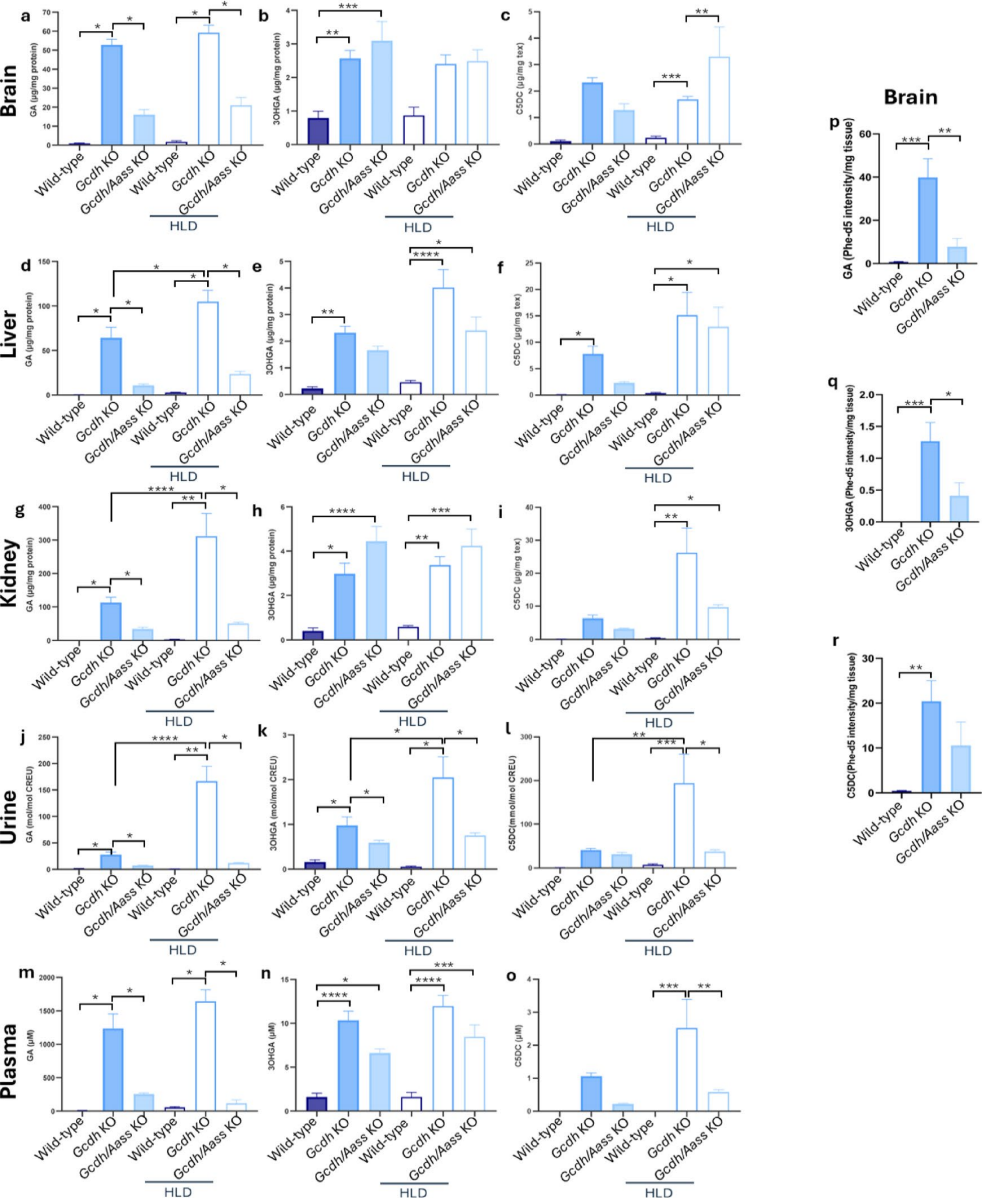
Metabolomic analysis in tissues and body fluids of 4-week-old mice. *Aass* knockout in GA1 mice restores the metabolic balance in GA1 mice under HLD. Brain GA (**a**), 3OHGA (**b**), and C5DC (**c**) levels, liver GA (**d**),3OHGA (**e**), and C5DC (**f**) levels, kidney GA (**g**), 3OHGA (**h**), and C5DC (**i**) levels, urine GA (**j**), 3OHGA (**k**), and C5DC (**l**) levels, and plasma GA (**m**), 3OHGA (**n**), and C5DC (**o**) levels were determined. Metabolomic level of 20-week-old mice brain GA (**p**), 3OHGA (**q**), and C5DC (**r**) levels. *n* = 6 for all groups (3 males, 3 females). There was no statistically significant difference in the metabolite levels of male and female animals, thus the values were combined. Statistical analysis was performed using one-way ANOVA followed by Tukey’s multiple comparisons test. Significance * p value < 0.05, ** for *p* ≤ 0.01, *** for *p* ≤ 0.001, **** for *p* ≤ 0.0001. Filled bar, standard diet; empty bar, HLD. Abbreviations: *Aass*, aminoadipate-semialdehyde synthase; CREU, creatinine; *Gcdh*, glutaryl-CoA dehydrogenase; HLD, high lysine diet; KO, knockout.

To further investigate the impact of induced AASS deficiency on the biochemical and neurobehavioral phenotype, we compared 4-week-old WT, *Gcdh* KO and *Gcdh/Aass* KO under standard diet. As expected, metabolomic studies in different tissues (brain, liver, heart, kidney) and body fluids (plasma, urine) revealed increased concentrations of GA (Fig. 5a, d,g, j,m), 3OHGA (Fig. 5b, e,h, k,n), and glutarylcarnitine (C5DC; Fig. 5c, f,i, l,o) in *Gcdh* KO mice compared to WT mice. Metabolomic analysis in brain samples of 20-week-old mice obtained similar results for GA (Fig. 5k). Biochemical analyses did not identify sex-specific differences. Therefore, subsequent experimental results of male and female mice were combined. In *Gcdh/Aass* KO mice, the biochemical phenotype was partially rescued compared to *Gcdh* KO mice. While concentrations of GA decreased to near-normal levels in *Gcdh/Aass* KO mice, this effect was less pronounced for 3OHGA (Fig. 5b, e,h, n), resembling the biochemical evaluation of *Gcdh* KO mice on lysine restriction^5^ and *Gcdh/Aass* KO mice on regular diet^33^. However, a more pronounced decrease of 3OHGA was seen in 20-week-old mice than early-stage measurements (Fig. 5l, Supplementary table).

Between the age of 9 to 20 weeks, mice were systematically studied using a standardized test pipeline that covers all clinically relevant organ systems, including multiple behavioral tests^34^. In comparison to WT mice and *Gcdh/Aass* KO mice, *Gcdh* KO mice presented with a decrease in rearing behavior (Fig. 4g), alteration of motor function with lower total distance travelled (Fig. 4h) and lower speed (open field, Fig. 4i), decreased grip strength in hind- and forelimbs (grip strength test; Fig. 4j), but unaltered reaction latencies towards heat thermal stimuli (hotplate test; Fig. 4k-n). Noteworthy, *Gcdh/Aass* KO mice could not be distinguished from WT mice in these comprehensive tests. The average activity of the *Gcdh* KO mice was slightly reduced and partially rescued in *Gcdh/Aass* KO mice (Supplementary Fig. 1a-c).

**Fig. 6:**
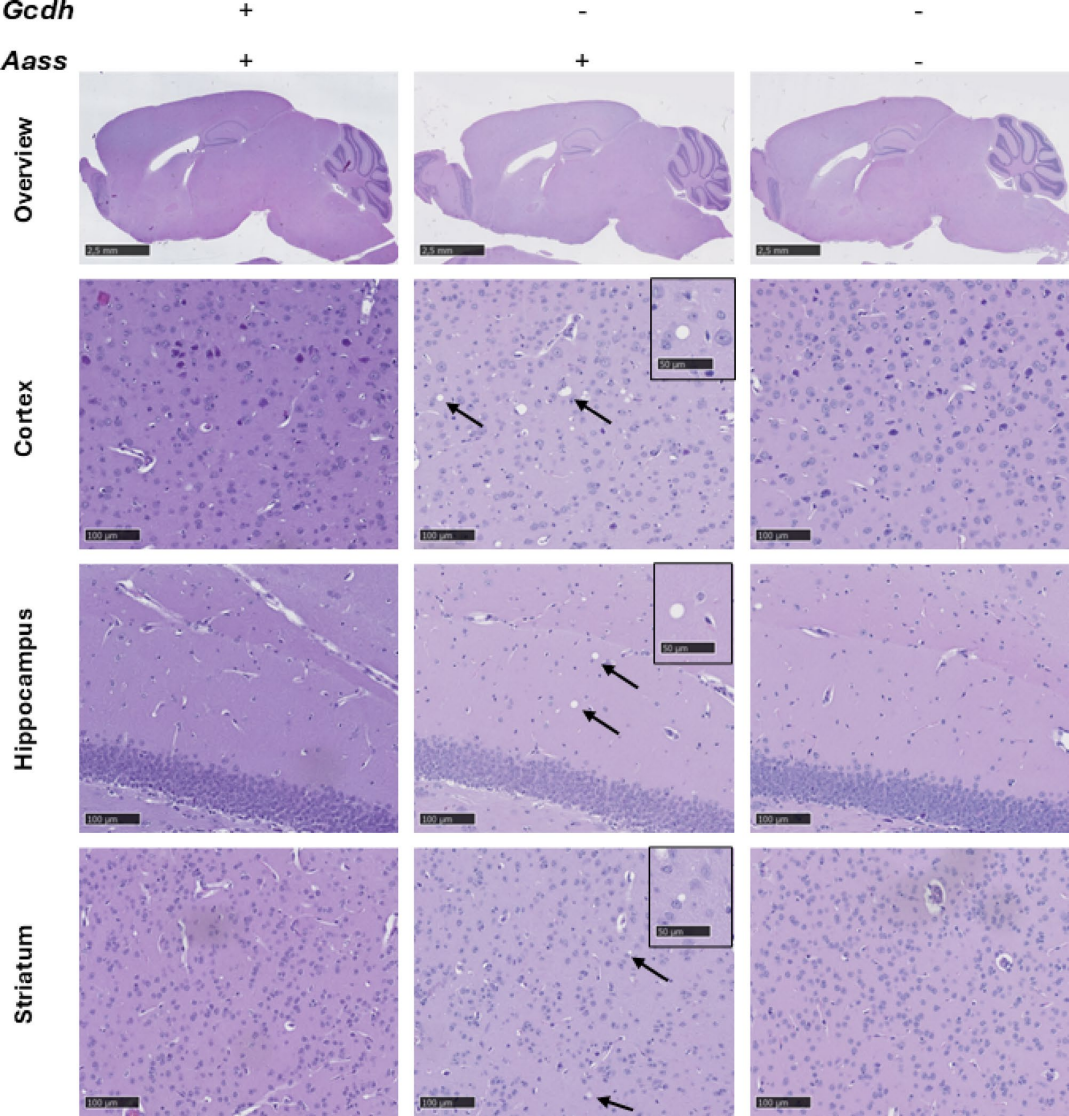
Representative images of hematoxylin and eosin-stained sagittal brain sections from a female control (wild-type), single (*Gcdh* KO) and double (*Gcdh*/*Aass* KO) knockout mice at 20 weeks of age under standard diet. The single knockout GA1 mice exhibited vacuoles (arrows; insets) in several brain regions (cortex, hippocampus and striatum), which were absent in the double knockout mice (rescue of the phenotype) (top panel: scale bar 2.5 mm, 1x lens, panels below: scale bar 100 μm, 20x lens, inset: scale bar 50 μm, 40x).

In line with the partially rescued biochemical phenotype of *Gcdh/Aass* KO mice, histological studies demonstrated that morphological changes in the brain of *Gcdh* KO mice were markedly reduced in *Gcdh/Aass* KO mice (Fig. 6). Hematoxylin and eosin staining indicated vacuoles in the cerebral cortex, hippocampus, striatum, and cerebellum in all (*n* = 10) analyzed brains of *Gcdh* KO mice without sex-specific differences. These morphological changes were found to be attenuated or even absent in *Gcdh/Aass* KO mice.

### Non-neurological phenotypes of *Gcdh* KO and *Gcdh/Aass* KO mice

**Fig. 7:**
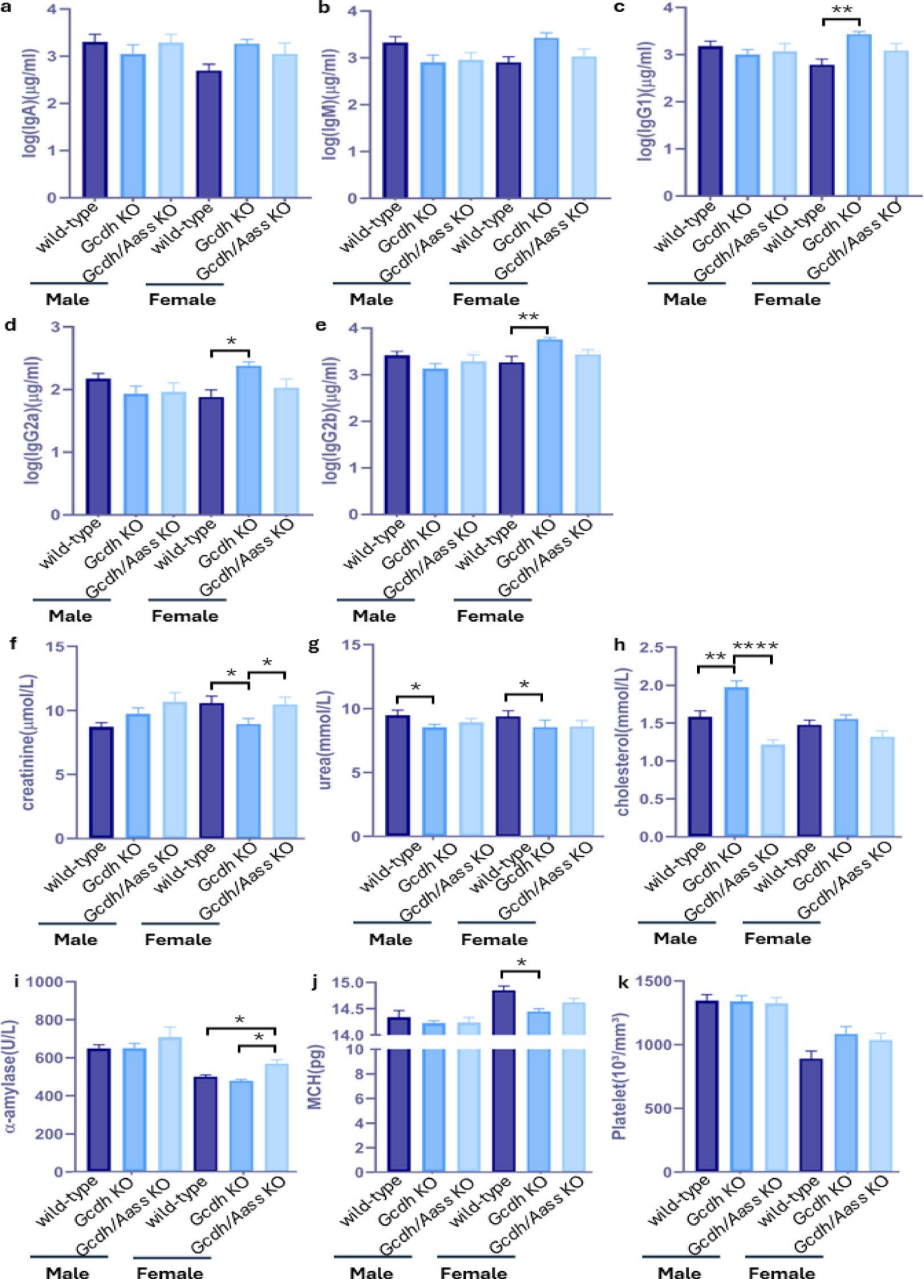
Immunological and clinical chemistry analysis of adult mice plasma. Multisport assay test includes (**a**) IgA levels, (**b**) IgM levels, (**c**) IgG1 levels, (**d**) IgG2a levels, (**e**) gG2b levels. Clinical chemistry examination includes (**f**) creatinine (µmol/L), (**g**) urea(mmol/L), (**h**) cholesterol (mmol/L), and (**i**) α-amylase (U/L). Hematological evaluation contains (**j**) MCH (pg) and (**k**) platelet count (10^3^/mm^3^). Statistical analysis was performed using one-way ANOVA followed by Tukey’s multiple comparisons test. Significance * p value < 0.05, ** for *p* ≤ 0.01, ** for *p* ≤ 0.01, *n* ≥ 10–15 for all groups. Abbreviations: *Aass*, aminoadipate-semialdehyde synthase; *Gcdh*, glutaryl-CoA dehydrogenase; KO, knockout.

**Fig. 8:**
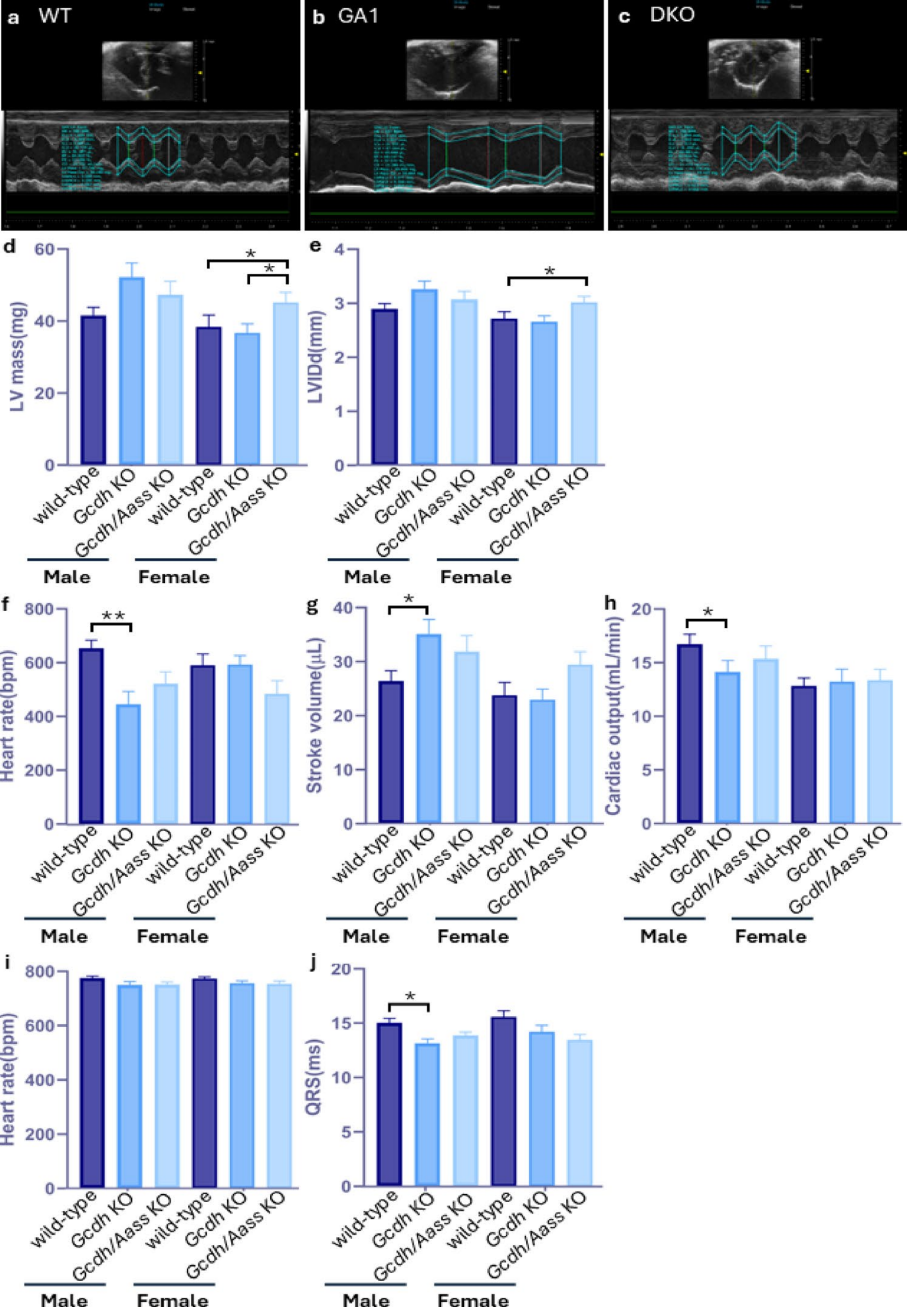
Cardiac phenotypes of adult *Gcdh KO* and *Gcdh/Aass* KO mice under standard diet. Transthoracic echocardiography imaging results in conscious mice include (**a**-**c**) representative short axis, m-mode echocardiogram images of male mice of the different backgrounds. (**d**) Left ventricle mass, (**e**) left ventricular internal diameter end-diastole (mm), (**f**) measured heart rate during the test (bpm), (**g**) stroke volume (µl), (**h**) cardiac output (ml/min) show disease specific changes in males. Electrocardiography in conscious mice (**i**) heart rate during the test (bpm), and (**j**) QRS complex length (ms). Statistical analysis was performed using one-way ANOVA followed by Tukey’s multiple comparisons test. Significance: * p value < 0.05, ** for *p* ≤ 0.01, *n* ≥ 10–15 for all groups. Abbreviations: *Aass*, aminoadipate-semialdehyde synthase; DKO, *Gcdh/Aass* KO; GA1, glutaric aciduria; *Gcdh*, glutaryl-CoA dehydrogenase; KO, knockout.

Since the enzymes of the lysine oxidation pathway are abundantly expressed in the body, we wondered whether GCDH deficiency might induce also non-neurological phenotypes. For this purpose, we first analyzed the body composition of *Gcdh* KO, *Gcdh/Aass* KO, and WT mice. Nuclear magnet resonance (NMR) imaging showed an overall similar body composition in all mice studied, irrespective of the underlying genotype or age (Supplementary Fig. 1d-g). We found a tendency to decreased overall lean body mass in 13-week-old male *Gcdh* KO mice but not in *Gcdh/Aass* KO mice and WT mice (Supplementary Fig. 1 d). The body (Supplementary Fig. 1 h) and organ weights (Supplementary Fig. 1i-k) exclusively showed expected sex-specific differences. No differences were detectable in bone mineralization (Supplementary Fig. 1l-m). Overall, although there are apparent differences in some parameters between groups, they are not statistically significant (*p* ≥ 0.05). Analysis of immunoglobulin subclasses showed equal results in male mice within the three groups studied. However, in female mice an immunological sex dimorphism affecting IgG1, IgG2a, and IgG2b was found in *Gcdh* KO mice but not in the *Gcdh/Aass* KO group (Fig. 7a-e). Additional biochemical tests (Fig. 7f-k) revealed slight differences in urea, creatinine, cholesterol levels, as well as in α-amylase, and mean corpuscular hemoglobin between the groups (Fig. 7f-k). Furthermore, functional cardiological tests (Fig. 8) revealed slight changes in left ventricular mass (Fig. 8d), heart rate (Fig. 8f), stroke volume (Fig. 8g), cardiac output (Fig. 8h), and QRS complex (Fig. 8j), among the studied groups. In summary, despite minor differences, the systematic evaluation of *Gcdh* KO and *Gcdh/Aass* KO mice did not reveal a significant non-neurological phenotype.

### Challenging *Gcdh* KO *and Gcdh/Aass* KO mice using HLD

To induce the severe acute phenotype and to evaluate the putative protective effect of *Aass* depletion, we challenged *Gcdh* KO and *Gcdh/Aass* KO mice with HLD as previously described^24^. Briefly, animals were either fed with regular chow containing 1.7% of lysine or chow containing 4.7% (w/w) of L-lysine for three days. Compared to mice on a standard diet, HLD led to an upregulation of *Aass* expression (Fig. 2a-d) in WT animals in liver and heart (for example in female liver 2.4-fold; *P* = 0.001). *Gcdh* expression was upregulated predominantly in liver and heart of WT animals (3.5-fold; *p* = 0.001; Fig. 2e-h). GCDH protein expression (Fig. 3a-d, i-l) was upregulated in all organs tested upon HLD in WT animals, whereas AASS protein expression (Fig. 2a-h) was enhanced only in liver and kidney of WT animals (Fig. 3f, g; kidney WT vs. WT with HLD: AASS 1.9-fold; GCDH 1.9-fold; *p* = 0.003). As expected, no upregulation of *Gcdh* mRNA or GCDH protein could be detected in *Gcdh* KO mice (Figs. 2e-h and 3a-d and i-l). Similarly, no upregulation of *Gcdh* mRNA or GCDH protein and *Aass* mRNA or AASS protein was detectable in *Gcdh/Aass* KO mice (Figs. 2a-d and 3a-h). Interestingly, *Aass* mRNA expression was upregulated in the liver *Gcdh* KO mice (Fig. 2b), as well as protein expression (in liver; Fig. 3b, f), pointing to a functional regulation of AASS expression in *Gcdh* KO mice. As expected, increased concentrations of GA, 3OHGA, and C5DC were detectable under HLD in urine and plasma of *Gcdh* KO mice (Fig. 5j-o in urine: GA 166.5 mol/mol creatinine vs. 27.8 mol/mol creatinine; *p* = 0.001; 3OHGA 2.05 mol/mol creatinine vs. 0.97 mol/mol creatinine; *p* = 0.001; C5DC 194.08 mol/mol creatinine vs. 7.04 mol/mol creatinine; *p* = 0.0006) as well as in all organs analyzed (Fig. 5a-i). In contrast, no significantly increased concentrations of GA and 3OHGA were detectable in body fluids and tissue homogenates of *Gcdh/Aass* KO mice under HLD. In *Gcdh/Aass* KO mice under HLD, C5DC levels were increased in the brain and liver but decreased in the kidney compared to WT (Fig. 5a-j). There was an improved overall clinical and neurological outcome, gain of weight, and lower rate of seizure-like behavior seen in *Gcdh/Aass* KO mice compared to *Gcdh* KO mice under HLD (Fig. 4a-f). This points to a beneficial and corrective effect of *Aass* depletion in GA1. Taken together, depletion or inhibition of AASS seems to be a promising option for the development of future therapies for GA1 as it leads to a reduced load of the body with toxic metabolites and an improved outcome in *Gcdh/Aass* KO mice compared to *Gcdh* KO mice.

## Discussion

This study aims to elucidate whether AASS could serve as a target for establishing a substrate reduction therapy for individuals with GA1. Investigating WT, *Gcdh* KO, and *Gcdh/Aass* KO mice under standard diet and HLD we demonstrated that the biochemical and clinical phenotype of *Gcdh* KO mice was partially rescued by knocking out *Aass*, supporting our hypothesis. This notion is supported by the findings that *Gcdh/Aass* KO mice (1) showed markedly reduced concentrations of neurotoxic dicarboxylic metabolites in tissue and body fluids, (2) had a neurobehavioral phenotype that could not be distinguished from WT mice in multiple behavioral tests, and (3) had a near-to-normal risk of developing a severe clinical phenotype induced by exposure to HLD compared to *Gcdh* KO mice.

The neurological phenotype of individuals with GA1 is thought to result from both acute and chronic effects exerted by the toxic metabolites GA, 3OHGA, and glutaryl-CoA, especially in the developing brain. The basal ganglia, particularly the putamen, exhibit high vulnerability during the first years of life. Energy demand, excitotoxic signaling via glutamatergic projections from the cortex and thalamus, metabolite-induced mitochondrial dysfunction, and the abundance of highly vulnerable medium-spiny neurons in the striatum are implicated in selective neuronal loss. Sequestration of glutaryl-CoA and its derivatives in the brain compartment^6^ and in mitochondria^8^ causes a glutaryl-CoA-induced inhibition of the 2-oxoglutarate dehydrogenase complex in the tricarboxylic acid cycle^5^. 3OHGA mediates activation of NMDA receptors and induction of excitotoxicity^35,36^. GA induces impairment of the transport of succinate and other energy-rich dicarboxylic acids from astrocytes to neurons^37^, and increased oxidative stress^38^. All events synergize and can further exacerbate under conditions of catabolism and metabolic stress. In this mechanistic multimodal model of neurodegeneration in GA1 converge systemic, local, age-dependent and incidental factors to provoke striatal injury during a vulnerable period of brain development^3^. Contrary to what is seen in patients, no pronounced neurological phenotype is present in the *Gcdh* KO mouse model unless it is induced by a HLD. This discrepancy likely reflects species‑specific differences in lysine metabolism and limits the full transferability of results generated in the animal model to human GA1^5^. although in mice and humans, accumulation of dicarboxylic toxic metabolites, particularly glutaryl-CoA, GA, and 3OHGA play a key role in pathogenicity and neuronal damage. As a consequence, currently recommended strategies for treatment aim to reduce the synthesis of glutaryl-CoA and its neurotoxic derivatives and to foster their detoxification in human patients^14,18^.

Low-lysine diet and dietary arginine supplementation aim to reduce the intracerebral concentrations of toxic metabolites. Carnitine supplementation aims to protect against the adverse effects of secondary carnitine depletion and facilitates the formation of non-toxic C5DC. Intermittent emergency treatments aim to restore anabolism and rapidly decrease toxic metabolite concentrations during events that are likely to induce catabolism, such as infectious disease. In individuals with GA1 who have been identified by NBS, have adhered to recommended therapy, and in whom treatment has been introduced before the manifestation of an irreversible neurological phenotype, the neurological outcome has been improved in the majority of early treated individuals^12,13^. The positive effect of this treatment was also confirmed in *Gcdh* KO mice^5,39^. However, about one-third of early treated patients do not yet have an improved neurological outcome. In addition, other disease manifestations, particularly chronic kidney disease and progressive periventricular white matter changes, do not seem to be impacted by current treatment strategies^12^. Therefore, safe and more effective therapies are needed to improve the long-term outcome of individuals with GA1. Substrate reduction therapy has long been considered as a potentially powerful strategy for GA1 to reduce flux through the lysine oxidation pathway and to decrease the formation of toxic metabolites, and to improve clinical outcome.

The establishment of a substrate reduction therapy for GA1 and other diseases is challenged by the search for a suitable target. The difficulty of this approach is documented by previous attempts that failed using DHTKD1 as a target^25,26^. In general, the druggable therapeutic targets should not have been associated with a severe clinical phenotype and should be effective to significantly reduce the formation of toxic metabolites. Lysine degradation occurs through two parallel pathways that converge later on^29^, i.e., the mitochondrial saccharopine pathway, initiated by the enzyme aminoadipic semialdehyde synthase (AASS; Fig. 1), and the peroxisomal pipecolate pathway. The latter remains incompletely characterized in humans. While in bacteria, two enzymes have been identified that catalyze the direct conversion of lysine to L-pipecolate^40^, these enzymatic steps have not yet been demonstrated in human metabolism. In humans, the first known enzyme in this pathway is pipecolate oxidase (PIPOX), which acts on L-pipecolate, as described previously in the lysine degradation pathway^29^. This gap of knowledge suggests that the upstream steps of L-pipecolate formation may differ fundamentally between species. Based on these uncertainties, researchers have proposed that in humans, L-pipecolate may not be generated via a conventional forward pathway but instead or in addition accumulates through a reverse flux when the mitochondrial saccharopine pathway is saturated^41^, underscoring the limited understanding of the pathway’s regulation and physiological relevance. Although treatment with clofibrate, an activator of the peroxisome proliferator-activated receptor α (PPARα), was shown to moderately reduce GA concentrations in liver and brain tissue of *Gcdh* KO mice through a yet unclear mechanism, currently we do not consider the pipecolate pathway a promising target for therapeutic intervention in GA1 due to the remaining ambiguity surrounding the function of the human L-pipecolate pathway and the lack of characterized enzymes upstream of PIPOX. Therefore, our search has been focused on the saccharopine pathway.

In the saccharopine pathway, L-lysine is first converted into saccharopine and then into α-aminoadipic semialdehyde, eventually leading to the formation of acetyl-CoA^29^. Besides GCDH in GA1 also other enzymes have been associated with rare metabolic diseases along this pathway, particularly inherited deficiency of ALDH7A1 (*ALDH7A1;* EC:1.2.1.31) causing pyridoxine-dependent epilepsy (PDE), a severe neurometabolic disease with early-onset infantile encephalopathy and impaired neurodevelopment^42^. Moreover, previous attempts to target DHTKD1 (*DHTKD1*; EC 1.2.4.2), i.e., the fifth step of the pathway, failed, since the 2-oxoglutarate dehydrogenase complex also contains a low substrate affinity for 2-oxoadipate and, therefore, knockout of *Dhtkd1* in mice did not rescue the biochemical and clinical phenotype of *Gcdh* KO mice^25,26^. Recently, certain gene variants of *DHTKD1* have been associated with Charcot-Marie-Tooth disease in a large Chinese pedigree^43^ and dementia with Lewy bodies in Japan^44^ doubting the safety of this approach. Finally, AASS (*AASS;* EC:1.5.1.8) and kynurenine/2-aminoadipate aminotransferase (AADAT/KAT2; *AADAT* EC 2.6.1.39) have not yet been associated with a significant clinical phenotype despite a measurable metabolic derangement. We decided to target AASS since this strategy could also be of therapeutic interest for individuals with PDE. AASS does not appear to cause severe phenotypes when partially inhibited. Individuals with hyperlysinemia due to AASS deficiency generally show mild or no symptoms^30,45^, supporting the enzyme’s safety profile as a target for pharmacological inhibition.

In the absence of enzymatic GCDH activity, glutaryl-CoA accumulates and is hydrolyzed to GA or further converted to 3OHGA via glutaconyl-CoA most likely involving side reactions of medium-chain acyl-CoA dehydrogenase and 3-methylglutaconyl-CoA hydratase^46^. While hepatic and renal tissues have been linked as sites of lysine catabolism, recent studies have examined whether AASS and the saccharopine pathway are also active in the brain. Notably, Sauer et al.^5^ and Sacksteder et al.^47^ reported that AASS expression is low to undetectable in adult murine brain tissues under basal conditions. However, expression of AASS can be induced specifically through challenging the L-lysine oxidation pathway with HLD, as we demonstrated in this study. Similarly, the entrapment hypothesis highlights the importance of accumulating neurotoxic metabolites in the brain^48^ and hence the relevance of a therapy that is able to approach the metabolic compartment of the brain. If the saccharopine pathway was functional in the brain, then its inhibition could play a crucial role in reducing local production of neurotoxic metabolites in GA1 and thus may build the basis for an innovative substrate reduction therapy. This study clearly shows that AASS is a promising therapeutic target for GA1 as its knockout resulted in a partial rescue of the biochemical and clinical phenotype usually seen in *Gcdh* KO mice and that it also has an impact on the brain compartment. This study supports earlier reports, showing that GA concentrations in urine and tissues, including the brain, are consistently higher than those of 3OHGA and more closely correlated with residual GCDH activity^49,50^. Furthermore, GA responds more pronouncedly to the induced AASS dysfunction compared to 3OHGA. Consequently, GA is considered a more reliable biomarker for disease monitoring and treatment response than 3OHGA. Finally, we observed a significant reduction in cerebral and hepatic GA concentrations in *Gcdh/Aass* KO mice, supporting the efficacy of AASS inhibition in decreasing the neurotoxic burden and in protecting against the manifestation of a severe acute phenotype. Moreover, the increase in C5DC in the brain and liver of *Gcdh/Aass* KO mice under HLD, likely reflecting partial metabolic rerouting, further supports the body’s physiologic ability to detoxify toxic metabolites of the lysine catabolic pathway. Our findings are consistent with those of Leandro et al.^33^, who also provided a biochemical validation of *AASS* KO in a GA1 cell line and mouse models. These data are supported by earlier biochemical studies that characterized AASS activity in different tissues and confirmed the enzyme’s central role in lysine catabolism^47^.

Although our study focused on genetic knockout of *Aass*, the concept of pharmacological AASS inhibition is increasingly relevant. At present, no clinically approved AASS inhibitors exist. Moreover, it would not be sufficient to inhibit only the LKR domain of AASS as this will not fully block enzymatic activity. Additionally, the inhibitor must cross the blood-brain barrier, to reach the highly impacted brain. Currently, several groups are working on the development of highly potent inhibitors of AASS, also within the CHARLIE consortium. For instance, a recent study aimed to achieve a therapeutic effect through an AAV‑delivered microRNA designed to silence AASS mRNA via RNA interference^51^.

Besides substrate reduction therapy, other therapeutic strategies are also tested for GA1. As discussed above, the marked cerebral reduction of GA (and 3OHGA) concentrations seems to be a suitable surrogate parameter to test the suitability of innovative therapies for GA1. Sauer et al.^6^ provided evidence that GA and 3OHGA exhibit limited permeability across the blood–brain barrier (BBB) owing to a lack of specific transporters, thereby restricting their systemic-to-cerebral transfer. This view was supported by neuropathological studies such as by Funk et al.^52^, showing that even patients with low urinary excretion of GA had unexpectedly high concentrations of these metabolites in the brain, suggesting *de novo* synthesis and subsequent accumulation in the brain. Earlier animal studies, including those by McMillan et al.^53^, described the fruit bat *Rousettus aegyptiacus*as an example of selective hepatic GCDH deficiency with preserved cerebral enzyme function. Despite excessive urinary excretion of GA and 3OHGA, these bats do not exhibit neurological symptoms, presumably because of absent cerebral metabolite accumulation. In contrast to the systemic approach proposed by Barzi et. al^27^., recent findings from Mateu-Bosch et al.^54^ suggests cerebral production of GA and 3OHGA. Thus, local inhibition of lysine degradation pathways plays a more decisive role in pathogenesis. Consequently, a dual-organ therapeutic strategy targeting AASS both in the liver and the CNS may offer the most comprehensive and robust substrate reduction therapy for GA1.

The investigation of the *Gcdh/Aass* KO mouse model clearly demonstrates the therapeutic potential of AASS inhibition. We observed normalized weight gain, reduced mortality, preserved striatal architecture, and near-complete behavioral rescue in *Gcdh/Aass* KO mice compared to *Gcdh* KO group. These effects were robust across both juvenile and adult time points and persisted under lysine-challenging conditions. Importantly, no overt toxicity or developmental abnormalities were observed in the *Gcdh/Aass* KO cohort.

Given the success of AASS inhibition in our GA1 model, we propose broader exploration of its utility in other lysine-related disorders. For instance, PDE also involves disrupted lysine catabolism. Studies by van Karnebeek et al.^55^ and Coughlin et al.^56^ suggest, that modifying lysine flux could alleviate symptoms in PDE. AASS inhibition may reduce upstream substrate levels and offer therapeutic synergy with existing pyridoxine (vitamin B_6_) supplementation. In conclusion, this study supports the safety and efficacy of AASS inhibition as a promising target for substrate reduction strategy in GA1. The *Gcdh/Aass* KO mouse model provides compelling preclinical evidence that pharmacologic targeting of AASS could transform the therapeutic landscape of this devastating disorder. Future studies should focus on compound optimization, delivery mechanisms, and long-term safety in humanized models.

## Methods

### Animal husbandry

Male and female WT, *Gcdh* KO, and *Gcdh*/*Aass* KO mice were all kept on a C57BL6 background. *Gcdh* KO were generated as previously described^24^. *Gcdh*/*Aass* KO animals were generated through backcrossing of *Aass* KO animals^33^ with *Gcdh* KO animals^27^. Animals were housed in 22 × 37 × 18 cm acrylic glass cages (up to five animals per cage) in an acclimatized room (22–26℃) with a 12-hour light/dark cycle. Animals had free access to water and either a standard (18,9% protein, 0.9% lysine; Rod18; Las Vendi) or high lysine diet (18,9% protein, 4.7% lysine; Rod18 with 39.26 g/kg L-lysine; Altromin International). Animals were acclimatized to housing conditions prior to the start of experiments, and cage bedding was changed weekly. Environmental enrichment (nesting material) was provided in all cages. At German Mouse Clinic (GMC), mice were maintained in IVC cages with water and standard mouse chow according to the directive 2010/63/EU, German laws and GMC housing conditions (www.mouseclinic.de).

The first animal cohort used for metabolic profiling was 4 weeks old and included WT, *Gcdh* KO, and *Gcdh/Aass* KO mice ± HLD for three days (*n* = 3 males and 3 females). Animals under standard or HLD were closely monitored using a mouse score sheet (see below) evaluating activity, weight, and seizure development. After three days ± HLD animals were euthanized using CO_2_ (no anesthesia; increasing concentration of CO_2_ from 10% to 30%). The group size was calculated with the G*Power program based on previous GA concentrations measured. The aim was to achieve a test strength of (1-β) > 0.8, which results from a significance level of *p* < 0.05. A second cohort of mice under standard diet conditions was used for systemic phenotyping at the GMC and contained WT (*n* = 15 males and 15 females), *Gcdh* KO (*n* = 15 males and 14 females), and *Gcdh/Aass* KO mice (*n* = 15 males and 12 females). The testing pipeline starts at 9 weeks of age. A group size of 15 animals each is sufficient to detect the desired difference in effect from a standard deviation (Cohen’s d = 2f) with a quality of 80% with the help of a double ANOVA at significance level 5% (www.mousephenotype.org).

Animals were allocated to experimental groups using simple randomization within genotype and sex strata. Outcome assessors and data analysts were blinded to group allocation, in experimental sessions not during monitoring, until the statistical analysis was completed to reduce bias.

### Genotyping

Ear punches from each mouse were collected for genotyping. These samples were mixed with a tissue lysis buffer and proteinase K and incubated at 60 °C for at least 2 h while shaking. Next, the samples were centrifuged and supernatant containing the DNA was collected, cleaned and used for genotyping PCR. The primers used for PCR were as follows: *Aass* (WT forward: GATATGCAGACAGGAGAGGTTAACC, KO forward: CCTTCAGGTTGAGAACTGGTGTT, WT and KO reverse: CAGAGCCAGAACAATAAGAAGACC), *Gcdh* (WT forward: CTTCCGTAACTACTGGCAGGAGCGG, WT reverse: AGCTCTCGGGTCAGAAGCCCATAGG, KO forward: GCGGTGGGCTCTATGGCTTCTGAGG, KO reverse: CCCAGAACTCAGGAGGAAGAGGCAG). The PCR conditions were as follows: initial denaturation (95 °C for 5 min), denaturation, annealing, and elongation (35 cycles; 95 °C for 30 s, 53 °C (*Aass* primers) or 69 °C (*Gcdh* primers) for 30 s, 72 °C for 30 s), final elongation (72 °C for 5 min). PCR products were run on an agarose gel (1%) and documented on a gel documentation system (PeqLab).

### Inclusion and exclusion criteria

Animals were included if they had the correct genotype and were in the correct age range (either 4- or 8-week-old at the experimental start). Animals were excluded if they did not survive the high-lysine diet challenge or showed signs of unrelated illness. All exclusions were pre-defined before analysis. No animals were excluded from the analysis for reasons other than those pre-specified above. Final numbers (n) for each experiment after exclusions were previously stated.

### Mouse score sheet

Mice were closely monitored during HLD, and a score was calculated to rate their general condition. For calculation, the weight of each mouse in the experiment was monitored during the three days challenge with HLD and the extent of weight loss was scored from 0 (no weight loss) to 3 (> 15% weight loss). Second, the occurrence, strength and persistence of seizures during the experiment was documented. Here, the score ranges from 0 (no seizures) to 3 (persistent tremor with prolonged periods of seizures). Third, changes in movement or normal behavior were recorded with 0 (normal behavior) up to 3 (no movement or grooming). All scores were then combined and averaged to give the overall condition score. If a score of 3 was reached in any score analyzed, animals were sacrificed.

### Behavioral testing

#### Open field test

The Open Field (OF) was assessed at 8 weeks of age and carried out as described previously^57^. The arena was made of transparent and infra-red light-permeable acrylic with a smooth floor (internal measurements: 45.5 × 45.5 × 39.5 cm). Illumination levels for the measurement were set at approx. 150 lx in the corners and 200 lx in the middle of the test arena. Data were recorded and analyzed using the ActiMot system (TSE, Germany) over a 20 min period.

#### Grip strength test

In the grip strength test the mouse grasps a grid mounted on a force sensor (Bioseb, Chaville, France). The mouse is allowed to catch the grid with either 2 or 4 paws. Then the maximum force applied by them before releasing the grid is recorded. Three trials were undertaken for each mouse and measurement within one minute. Mean values were used for statistical analysis using a linear model with body weight as covariate.

#### Hotplate test

In the hotplate test the mice were placed on a metal surface maintained at 52 ± 0.2 °C surrounded by a plexiglass wall (Hot plate system TSE GMBH, Germany). Mice remained on the plate until they performed the second of three behaviors regarded as indicative of nociception: hind paw lick, hind paw shake/flutter or jumping during the maximum testing time of 30 s.

### Nuclear magnetic resonance (NMR) for body composition analysis

Nuclear Magnetic Resonance (NMR) spectroscopy was employed to determine fat mass, lean body mass, and total body water content in mice. Measurements were performed using a Bruker Minispec LF50 body composition analyzer, which provides precise, non-invasive quantification of body composition in live animals. Mice were individually placed into a restraining tube without anesthesia to minimize stress and movement artifacts during scanning. The whole-body NMR scan was conducted using a 10 MHz resonance frequency optimized for small animal analysis. The instrument automatically differentiated between fat, lean tissue, and free body water based on hydrogen proton relaxation times.

### Dual-energy X-ray absorptiometry (DXA)

DXA was performed with a Faxitron UltraFocus device (Faxitron Bioptics, LLC), to analyze whole-mouse (excluding skull) bone mineral content (BMC) and density (BMD).

### Indirect calorimetrics

At the age of 11 weeks, home cage locomotor activity monitoring, gas exchange (oxygen consumption and carbon dioxide production), heat production and substrate utilization were measured for individually caged mice by indirect calorimetry in metabolic home cages (TSE Systems GmbH, Berlin, Germany,  https://www.mousephenotype.org/impress/ProcedureInfo?action=list&procID=852&pipeID=7). The measurement commenced five hours before lights off and finished four hours after lights-on the next morning (21 h in total)^58^.

### Clinical chemistry analysis

Blood samples were collected under isoflurane anesthesia via retrobulbar puncture in Li-heparin coated sample tubes. Samples were stored at room temperature for a maximum of 2 h before plasma was separated by centrifugation (4.500×g for 10 min at 8 °C) and analyzed using an Olympus AU480 Chemistry Analyzer for markers of liver and kidney function, including urea, α-amylase, creatinine, glucose, and lipid profiles^59^.

### Hematology

An aliquot of 50 µl whole blood was collected in an 50 µl end-to-end capillary and diluted 1:7 with Sysmex Cell Pack DCL dilution buffer followed analysis of blood cell counts using the Sysmex XN 1000 V analyzer with preset mouse settings applying the pre-dilution mode.

### Immunoglobulin levels in mouse plasma

Immunoglobulin subtypes in plasma samples were quantified using a multiplex MSD immunoassay platform (Meso Scale Discovery, Rockville, MD, USA). Assays were performed according to the manufacturer’s instructions provided in the kit insert. Briefly, plasma samples were diluted 1:10 in diluent buffer. Following incubation, a detection antibody was added, and the plates were read using the MESO^®^ QuickPlex SQ 120MM instrument.

### Electrocardiogram (ECG) and transthoracic echocardiogram (TTE)

ECG recordings were performed in conscious mice using a ECGenie™ System (Mouse Specifics, Inc.). Data was collected to assess electrical conduction alterations based on peak detection and interval lengths. TTE was conducted using a Vevo^®^3100 system (Visual Sonics, Fuji Film) in conscious mice. M-mode imaging at the level of the papillary muscles was performed to measure left ventricular morphology and calculate ejection fraction (LVEF), fractional shortening, and cardiac output with the Teichholz formula^60^.

### RT-qPCR

To detect the presence of *Gcdh* and *Aass* transcripts in mouse brain, kidney, heart and liver, RT–qPCR was performed with total RNA isolated from these tissues using Trizol^™^ reagent according to the manufacturer’s instructions. RNA concentrations were evaluated at 260 and 280 nm with a NanoDroplite (Thermo Scientific). First-strand cDNA was synthesized from 1 µg of RNA using the cDNA Reverse Superscript III Transcription Kit (Thermo Fisher) with a random hexamer primer. Luna Script master mix (M3003, NEB) was used to detect mouse *Gcdh* mRNA (forward primer: TCGGGGCTTCATACTGGAGA, reverse primer: GCACATTCTCCTCAGGCACT) and *Aass* mRNA (forward primer: GTCTTCACAGGGACTGGCAA, reverse primer: GCGACTTAACACCGTCCCAT) normalized to *Gapdh* expression (forward primer: TGCACCACCAACTGCTTAG, reverse primer: GGATGCAGGGATGATGTTC). PCR was performed according to the following protocol: 95˚C for 1 min as initial denaturation and 95˚C for 30 s, 60˚C for 30 s for 35 cycles. Target transcripts relative expression levels were determined by the ΔΔCt method, using WT mice at each time point as calibrators.

### Western blot

Protein content in samples was quantified according to the method of Lowry using bovine serum albumin as standard. Organs were disrupted using a tissue homogenizer and homogenized in RIPA buffer with protease inhibitors (cOmplete^™^ Mini, EDTA-free Protease Inhibitor Cocktail, Roche). The homogenate was centrifuged for 20 min at 11.000×rpm at 4 °C in an Eppendorf centrifuge. 40 µg of total protein was prepared in 6x sodium dodecyl sulfate (SDS) sample buffer (0.5 mol/L Tris–HCl, pH 6.8, 10% glycerol, 1%SDS and 0.01% bromophenol blue) and separated on 10% SDS-acrylamide gels at 100 V followed by blotting to a PVDF membrane (120 mA for 90 min). Membranes were blocked for 1 h at RT in blocking solution (5% BSA[w/v]/TBST buffer) and protein expression was detected with an antibody against GCDH (Atlas, HPA 043252; diluted 1:10.000 in 2.5% BSA[w/v]/TBST buffer), AASS (Atlas, HPA 020734; diluted 1:4.000 diluted in blocking solution), or GAPDH (Invitrogen, AM4300; diluted 1:3.000 in blocking buffer) over night at 4 °C. After washing with TBST (3 × 5 min, RT), the secondary antibody was applied (anti-rabbit: Cell Signaling Technology, 7074; anti-mouse: Cell Signaling Technology, 7076 S) for 1 h at RT. After a final washing (TBST, 3 × 5 min, RT), ECL was used for detection on a Fusion system. GAPDH was used for normalization.

### Metabolomics analysis

Quantification of GA, 3OHGA, and glutarylcarnitine in liver, brain, kidney, plasma, and urine of mice was performed by Liquid Chromatography coupled with Tandem Mass Spectrometry (LC-MS/MS) as well as urine creatinine. LC-MS/MS is used to separate, detect, and quantify a range of metabolites in tissue and body fluids samples. Tissue organic acid was determined using the same analysis procedure with a homogenate in water containing approximately 30 mg wet weight as matrix for samples extracted at 4 weeks old, and 200 mg wet weight as matrix for samples extracted at 20 weeks old. Values were normalized to protein concentration in tissues, and to creatinine in urine.

### Histological analysis of brain tissue

Tissues from WT, *Gcdh* KO, and *Gcdh/Aass* KO mice under standard diet (aged 20 weeks, *n* = 5 per sex per genotype) were fixed after CO_2_ euthanasia (no anesthesia; increasing concentration of CO_2_ from 10% to 30%) in neutral-buffered formalin, processed, paraffin-embedded, sectioned at 3 μm and stained with standard hematoxylin and eosin (HE) for blinded high-throughput histopathological evaluation, as previously described^34^. Brain HE-stained slides were imaged with a NanoZoomer S60 digital scanner (Hamamatsu, Japan) and examined with NDP.View2 Software.

### Statistical analysis

All statistical analyses were performed using GraphPad Prism version 8.0 (GraphPad Software, San Diego, CA, USA). For normally distributed data with equal variances, comparisons between more than two groups were made using one-way ANOVA followed by Tukey’s or Bonferroni post hoc tests as appropriate. Where necessary, data were log-transformed to meet test assumptions. All measurements were expressed as mean ± standard deviation and analyzed using one-way ANOVA unless stated otherwise. Statistically significant differences were considered if *p* < 0.05.

## Supplementary Information

Below is the link to the electronic supplementary material.

Supplementary Material 1

Supplementary Material 2

## Data Availability

The datasets generated during and/or analyzed during the current study are available from the corresponding author upon reasonable request. The original Western Blot membranes are included into Supplementary data. Raw data of metabolite measurements in the brain of 20-weeks-old mice are included into the Supplementary table.

## Abbreviations

3OHGA: 3-hydroxyglutaric acid
AASS: Aminoadipate-semialdehyde synthase
CoA: Coenzyme A
DHTKD1: Dehydrogenase E1 and transketolase domain‑containing protein 1
FAD: Flavin adenine dinucleotide
GA: Glutaric acid
GA1: Glutaric aciduria type 1
GCDH: Glutaryl-CoA dehydrogenase
HE: High excreter
HLD: High-lysine diet
KO: Knockout
LE: Low excreter
LOR: Lysine-2-oxoglutarate reductase
SDH: Saccharopine dehydrogenase
NBS: Newborn screening
WT: Wild-type

## References

[1] Paria, P. et al. Identification of novel pathogenic variants in the GCDH gene and assessment of neurodevelopmental outcomes in 24 children with glutaric aciduria type 1. *Eur. J. Paediatr. Neurol.* **39**, 49–58 (2022). 35662016 10.1016/j.ejpn.2022.05.005

[2] Kolker, S. et al. Diagnosis and management of glutaric aciduria type I–revised recommendations. *J. Inherit. Metab. Dis.* **34** (3), 677–694 (2011). 21431622 10.1007/s10545-011-9289-5 PMC3109243

[3] Dimitrov, B. et al. Organic acidurias: Major gaps, new challenges, and a yet unfulfilled promise. *J. Inherit. Metab. Dis.* **44** (1), 9–21 (2021). 32412122 10.1002/jimd.12254

[4] Sauer, S. W. et al. Bioenergetics in glutaryl-coenzyme A dehydrogenase deficiency: a role for glutaryl-coenzyme A. *J. Biol. Chem.* **280** (23), 21830–21836 (2005). 15840571 10.1074/jbc.M502845200

[5] Sauer, S. W. et al. Therapeutic modulation of cerebral L-lysine metabolism in a mouse model for glutaric aciduria type I. *Brain* **134** (Pt 1), 157–170 (2011). 20923787 10.1093/brain/awq269

[6] Sauer, S. W. et al. Intracerebral accumulation of glutaric and 3-hydroxyglutaric acids secondary to limited flux across the blood-brain barrier constitute a biochemical risk factor for neurodegeneration in glutaryl-CoA dehydrogenase deficiency. *J. Neurochem*. **97** (3), 899–910 (2006). 16573641 10.1111/j.1471-4159.2006.03813.x

[7] Sauer, S. W. et al. Glutaric aciduria type I and methylmalonic aciduria: simulation of cerebral import and export of accumulating neurotoxic dicarboxylic acids in in vitro models of the blood-brain barrier and the choroid plexus. *Biochim. Biophys. Acta*. **1802** (6), 552–560 (2010). 20302929 10.1016/j.bbadis.2010.03.003

[8] Mitchell, G. A. et al. Hereditary and acquired diseases of acyl-coenzyme A metabolism. *Mol. Genet. Metab.* **94** (1), 4–15 (2008). 18337138 10.1016/j.ymgme.2007.12.005

[9] Wagner, G. R. et al. A Class of Reactive Acyl-CoA Species Reveals the Non-enzymatic Origins of Protein Acylation. *Cell. Metab.* **25** (4), 823–837e8 (2017). 28380375 10.1016/j.cmet.2017.03.006 PMC5399522

[10] Schmiesing, J. et al. Disease-Linked Glutarylation Impairs Function and Interactions of Mitochondrial Proteins and Contributes to Mitochondrial Heterogeneity. *Cell. Rep.* **24** (11), 2946–2956 (2018). 30208319 10.1016/j.celrep.2018.08.014

[11] Strauss, K. A. et al. Glutaric acidemia type 1: Treatment and outcome of 168 patients over three decades. *Mol. Genet. Metab.* **131** (3), 325–340 (2020). 33069577 10.1016/j.ymgme.2020.09.007

[12] Boy, N. et al. Newborn screening: A disease-changing intervention for glutaric aciduria type 1. *Ann. Neurol.* **83** (5), 970–979 (2018). 29665094 10.1002/ana.25233

[13] Boy, N. et al. Impact of newborn screening and quality of therapy on the neurological outcome in glutaric aciduria type 1: a meta-analysis. *Genet. Med.* **23** (1), 13–21 (2021). 32981931 10.1038/s41436-020-00971-4 PMC7790745

[14] Boy, N. et al. Recommendations for diagnosing and managing individuals with glutaric aciduria type 1: Third revision. *J. Inherit. Metab. Dis.* **46** (3), 482–519 (2023). 36221165 10.1002/jimd.12566

[15] Gordon, N. Glutaric aciduria types I and II. *Brain Dev.* **28** (3), 136–140 (2006). 16368216 10.1016/j.braindev.2005.06.010

[16] Strauss, K. A. et al. Safety, efficacy and physiological actions of a lysine-free, arginine-rich formula to treat glutaryl-CoA dehydrogenase deficiency: focus on cerebral amino acid influx. *Mol. Genet. Metab.* **104** (1–2), 93–106 (2011). 21820344 10.1016/j.ymgme.2011.07.003

[17] Yoldas Celik, M. et al. Glutaric aciduria type 1: Insights into diagnosis and neurogenetic outcomes. *Eur. J. Pediatr.* **184** (1), 72 (2024). 39658645 10.1007/s00431-024-05907-7

[18] Heringer, J. et al. Use of guidelines improves the neurological outcome in glutaric aciduria type I. *Ann. Neurol.* **68** (5), 743–752 (2010). 21031586 10.1002/ana.22095

[19] Harting, I. et al. Dynamic changes of striatal and extrastriatal abnormalities in glutaric aciduria type I. *Brain* **132** (Pt 7), 1764–1782 (2009). 19433437 10.1093/brain/awp112

[20] Singanamalla, B. et al. *The challenge of severe acute malnutrition in inborn errors of metabolism: Does medical food alone suffice?*. *J. Pediatr. Genet.* **12**(2), 175–178 (2023). 37090831 10.1055/s-0041-1739288 PMC10118697

[21] Mateu-Bosch, A. et al. Modeling glutaric aciduria type I in human neuroblastoma cells recapitulates neuronal damage that can be rescued by gene replacement. *Gene Ther.* **31**(1–2), 12–18 (2024). 37985879 10.1038/s41434-023-00428-8

[22] Das, A. M. et al. Diagnosis, treatment, management and monitoring of patients with tyrosinaemia type 1: Consensus group recommendations from the German-speaking countries. *J. Inherit. Metab. Dis.* **48** (1), e12824 (2025). 39676394 10.1002/jimd.12824 PMC11647197

[23] Torralba-Cabeza, M. A. et al. Recommendations for oral treatment for adult patients with type 1 Gaucher disease. *Rev. Clin. Esp. (Barc)*. **222** (9), 529–542 (2022). 35676195 10.1016/j.rceng.2022.02.008

[24] Sauer, S. W. et al. Multifactorial modulation of susceptibility to l-lysine in an animal model of glutaric aciduria type I. *Biochim. Biophys. Acta*. **1852** (5), 768–777 (2015). 25558815 10.1016/j.bbadis.2014.12.022

[25] Biagosch, C. et al. Elevated glutaric acid levels in Dhtkd1-/Gcdh- double knockout mice challenge our current understanding of lysine metabolism. *Biochim. Biophys. Acta Mol. Basis Dis.* **1863** (9), 2220–2228 (2017). 28545977 10.1016/j.bbadis.2017.05.018

[26] Leandro, J. et al. DHTKD1 and OGDH display substrate overlap in cultured cells and form a hybrid 2-oxo acid dehydrogenase complex in vivo. *Hum. Mol. Genet.* **29** (7), 1168–1179 (2020). 32160276 10.1093/hmg/ddaa037 PMC7206849

[27] Barzi, M. et al. Rescue of glutaric aciduria type i in mice by liver-directed therapies. *Sci. Transl. Med.* **15**(692), eadf4086 (2023). 37075130 10.1126/scitranslmed.adf4086 PMC10676743

[28] Sander, M., Houten1, Heleen te Brinke1 & Alida, S. Denis1 C Knegt3, Johannis BC de Klerk4, and J.H. Persephone Augoustides-Savvopoulou5, Matthias R Baumgartner6, Turgay Coşkun8, Johannes Zschocke9, Jörn Oliver Sass7,10, Bwee Tien Poll-The2, Ronald JA Wanders1,2 and Marinus Duran1,2, *Genetic basis of hyperlysinemia.* Orphanet Journal of Rare Diseases 8:57. (2013). 10.1186/1750-1172-8-57 PMC3626681 23570448

[29] Leandro, J. & Houten, S. M. The lysine degradation pathway: Subcellular compartmentalization and enzyme deficiencies. *Mol. Genet. Metab.* **131** (1–2), 14–22 (2020). 32768327 10.1016/j.ymgme.2020.07.010

[30] DANCIS, J. et al. The Prognosis of Hyperlysinemia: An Interim Report. *Am. J. Hum. Genet.* **35**, 438–442 (1983). 6407303 PMC1685659

[31] Justo, R. et al. Gender dimorphism in rat liver mitochondrial oxidative metabolism and biogenesis. *Am. J. Physiol. Cell. Physiol.* **289** (2), C372–C378 (2005). 15800054 10.1152/ajpcell.00035.2005

[32] Dearden, L., Bouret, S. G. & Ozanne, S. E. Sex and gender differences in developmental programming of metabolism. *Mol. Metab.* **15**, 8–19 (2018). 29773464 10.1016/j.molmet.2018.04.007 PMC6066743

[33] Leandro, J. et al. Deletion of 2-aminoadipic semialdehyde synthase limits metabolite accumulation in cell and mouse models for glutaric aciduria type 1. *J. Inherit. Metab. Dis.* **43** (6), 1154–1164 (2020). 32567100 10.1002/jimd.12276

[34] Fuchs, H. et al. Understanding gene functions and disease mechanisms: Phenotyping pipelines in the German Mouse Clinic. *Behav. Brain Res.* **352**, 187–196 (2018). 28966146 10.1016/j.bbr.2017.09.048

[35] Kolker, S. et al. Pathomechanisms of neurodegeneration in glutaryl-CoA dehydrogenase deficiency. *Ann. Neurol.* **55** (1), 7–12 (2004). 14705106 10.1002/ana.10784

[36] Kolker, S. et al. Excitotoxicity and bioenergetics in glutaryl-CoA dehydrogenase deficiency. *J. Inherit. Metab. Dis.* **27** (6), 805–812 (2004). 15505385 10.1023/B:BOLI.0000045762.37248.28

[37] Lamp, J. et al. Glutaric aciduria type 1 metabolites impair the succinate transport from astrocytic to neuronal cells. *J. Biol. Chem.* **286** (20), 17777–17784 (2011). 21454630 10.1074/jbc.M111.232744 PMC3093853

[38] Wajner, M. Neurological manifestations of organic acidurias. *Nat. Rev. Neurol.* **15**(5), 253–271 (2019). 30914790 10.1038/s41582-019-0161-9

[39] Zinnanti, W. J. et al. Mechanism of age-dependent susceptibility and novel treatment strategy in glutaric acidemia type I. *J. Clin. Invest.* **117** (11), 3258–3270 (2007). 17932566 10.1172/JCI31617 PMC2000809

[40] Revelles, O., Wittich, R. M. & Ramos, J. L. Identification of the initial steps in D-lysine catabolism in Pseudomonas putida. *J. Bacteriol.* **189** (7), 2787–2792 (2007). 17259313 10.1128/JB.01538-06 PMC1855791

[41] Struys, E. A. & Jakobs, C. Metabolism of lysine in alpha-aminoadipic semialdehyde dehydrogenase-deficient fibroblasts: evidence for an alternative pathway of pipecolic acid formation. *FEBS Lett.* **584** (1), 181–186 (2010). 19932104 10.1016/j.febslet.2009.11.055

[42] Korasick, D. A., Tanner, J. J. & Henzl, M. T. Impact of disease-Linked mutations targeting the oligomerization interfaces of aldehyde dehydrogenase 7A1. *Chem. Biol. Interact.* **276**, 31–39 (2017). 28087462 10.1016/j.cbi.2017.01.002 PMC5503811

[43] Xu, W. Y. et al. A nonsense mutation in DHTKD1 causes Charcot-Marie-Tooth disease type 2 in a large Chinese pedigree. *Am. J. Hum. Genet.* **91** (6), 1088–1094 (2012). 23141294 10.1016/j.ajhg.2012.09.018 PMC3516600

[44] Mitsumori, R. et al. A genome-wide association study identifies a novel East Asian-specific locus for dementia with Lewy bodies in Japanese subjects. *Mol. Med.* **31** (1), 87 (2025). 40045203 10.1186/s10020-025-01115-7 PMC11884146

[45] Yeganeh, M. et al. A case of hyperlysinemia identified by urine newborn screening. *JIMD Rep.* **64** (6), 440–445 (2023). 37927488 10.1002/jmd2.12399 PMC10623103

[46] Peters, V. et al. Formation of 3-hydroxyglutaric acid in glutaric aciduria type I: in vitro participation of medium chain acyl-CoA dehydrogenase. *JIMD Rep.* **47** (1), 30–34 (2019). 31240164 10.1002/jmd2.12026 PMC6498835

[47] Sacksteder, K. A. et al. Identification of the alpha-aminoadipic semialdehyde synthase gene, which is defective in familial hyperlysinemia. *Am. J. Hum. Genet.* **66** (6), 1736–1743 (2000). 10775527 10.1086/302919 PMC1378037

[48] Hallen, A., Jamie, J. F. & Cooper, A. J. Lysine metabolism in mammalian brain: an update on the importance of recent discoveries. *Amino Acids*. **45** (6), 1249–1272 (2013). 24043460 10.1007/s00726-013-1590-1 PMC3838446

[49] Kolker, S. et al. Natural history, outcome, and treatment efficacy in children and adults with glutaryl-CoA dehydrogenase deficiency. *Pediatr. Res.* **59** (6), 840–847 (2006). 16641220 10.1203/01.pdr.0000219387.79887.86

[50] Strauss, K. A. et al. Type I glutaric aciduria, part 1: natural history of 77 patients. *Am. J. Med. Genet. C Semin Med. Genet.* **121C** (1), 38–52 (2003). 12888985 10.1002/ajmg.c.20007

[51] Segur-Bailach, E. et al. Therapeutic AASS inhibition by AAV-miRNA rescues glutaric aciduria type I severe phenotype in mice. *Mol. Ther.* **33** (10), 4820–4833 (2025). 40682274 10.1016/j.ymthe.2025.07.022 PMC12848188

[52] Funk, C. B. R. et al. Neuropathological, biochemical and molecular findings in a glutaric acidemia type 1 cohort. *Brain* **128** (4), 711–722 (2005). 15689364 10.1093/brain/awh401

[53] McMillan, T. A. et al. Conservation of central nervous system glutaryl-coenzyme A dehydrogenase in fruit-eating bats with glutaric aciduria and deficient hepatic glutaryl-coenzyme A dehydrogenase. *J. Biol. Chem.* **263** (33), 17258–17261 (1988). 3182847

[54] Mateu-Bosch, A. et al. Systemic delivery of AAV-GCDH ameliorates HLD-induced phenotype in a glutaric aciduria type I mouse model. *Mol. Ther. Methods Clin. Dev.* **32** (3), 101276 (2024). 38983872 10.1016/j.omtm.2024.101276 PMC11231595

[55] van Karnebeek, C. D. et al. Lysine-Restricted Diet as Adjunct Therapy for Pyridoxine-Dependent Epilepsy: The PDE Consortium Consensus Recommendations. *JIMD Rep.* **15**, 1–11 (2014). 24748525 10.1007/8904_2014_296 PMC4270869

[56] Coughlin, C. R. et al. Triple therapy with pyridoxine, arginine supplementation and dietary lysine restriction in pyridoxine-dependent epilepsy: Neurodevelopmental outcome. *Mol. Genet. Metab.* **116**(1–2), 35–43 (2015). 26026794 10.1016/j.ymgme.2015.05.011

[57] Lillian Garrett1. D Chichung Lie1, Martin Hrabé de Angelis2,3,5, Wolfgang Wurst1,4,6,7* and Sabine M Hölter1,3,4*, *Voluntary wheel running in mice increases the rate of neurogenesis without affecting anxiety-related behaviour in single tests.* Garrett et al. BMC Neuroscience, (2012). 10.1186/1471-2202-13-61 PMC3504529 22682077

[58] Rozman, J., Klingenspor, M. & Hrabe de Angelis, M. A review of standardized metabolic phenotyping of animal models. *Mamm. Genome*. **25** (9–10), 497–507 (2014). 25199945 10.1007/s00335-014-9532-0

[59] Rathkolb, B. et al. Clinical Chemistry and Other Laboratory Tests on Mouse Plasma or Serum. *Curr. Protoc. Mouse Biol.* **3** (2), 69–100 (2013). 26069059 10.1002/9780470942390.mo130043

[60] LOUIS EVAN TEICHHOLZ, M., FACC THOMAS KREULEN, M. D., FACC+, M. I. C. H. A. E. L. V. & HERMAN, MD, FACC’ RICHARD GORLIN, M. D. Problems in Echocardiographic Volume Determinations: Echocardiographic-Angiographic Correlations in the Presence or Absence of Asynergy. *American J. Cardiol.* **37**, 7–11 (1976). 10.1016/0002-9149(76)90491-4 1244736

