# Supplementary material for: Aminoadipate-semialdehyde synthase, a potential target for substrate reduction therapy in glutaric aciduria type 1
Source: Sci Rep. 2026 Mar 31;16:10995. doi: 10.1038/s41598-026-44377-9 (PMC13043701; doi:10.1038/s41598-026-44377-9)
Supplement: Supplementary file 2 — Supplementary Material 2 [file 41598_2026_44377_MOESM2_ESM.docx]

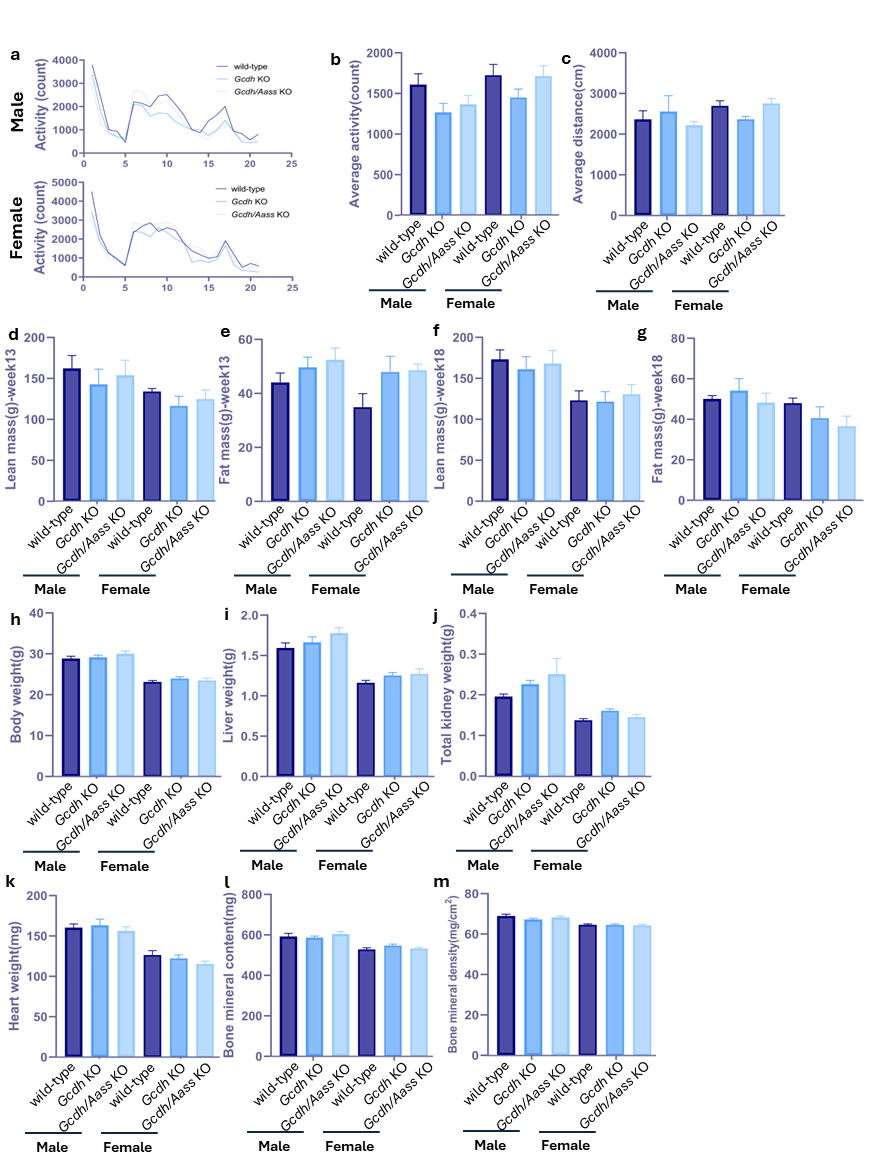
**Supplementary figure 1.** Body composition analysis under standard diet. Nuclear magnetic resonance (NMR) measurements were performed at 13 and 18 weeks of age. (a) lean mass (in g) at week 13, (b) fat mass (in g) at week 13, (c) lean mass (in g) at week 18, and (d) fat mass (in g) at week 18. (e) determination of total body weight (in g) at week 18, the weight of several organs at 20 weeks ((f) liver weight (in g), (g) total kidney weight (in g), (h) heart weight (in g), (i) bone mineral content (in mg), and (j) bone mineral density (in mg/cm²) show no significant changes between the genetic backgrounds but differ between sexes. Indirect calorimetric analysis includes the measurement of (k) mice activity per count over 21 hours, (l) mice average activity, and (m) mice average distance traveled (in cm). Statistical analysis was performed using non-parametric one-way ANOVA did not reveal significant differences between the groups for any of the tested parameters. n≥ 10-15 for all groups. Abbreviations: *Aass*, aminoadipate-semialdehyde synthetase; *Gcdh*, glutaryl-CoA dehydrogenase; KO, knockout.

**Western Blot full membranes (raw data; uncropped)**

Western Blot membranes detecting AASS, GCDH, and GAPDH protein in brain, liver, kidney, and heart of male and female mice of the different genotypes with and without HLD. GAPDH was used as loading control.

1.
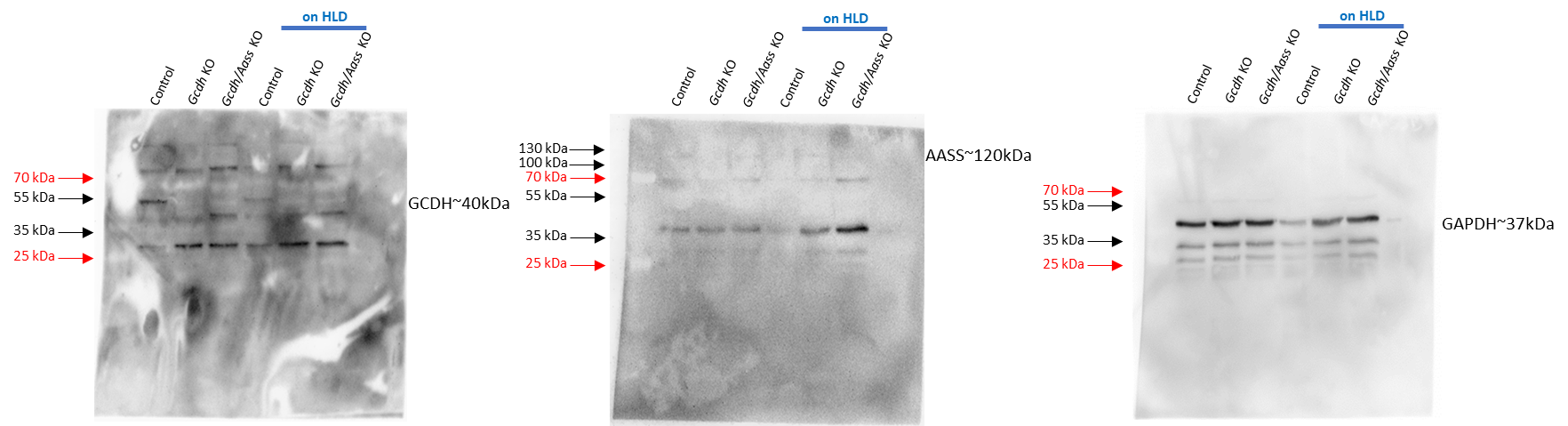
**Brain male**
2.
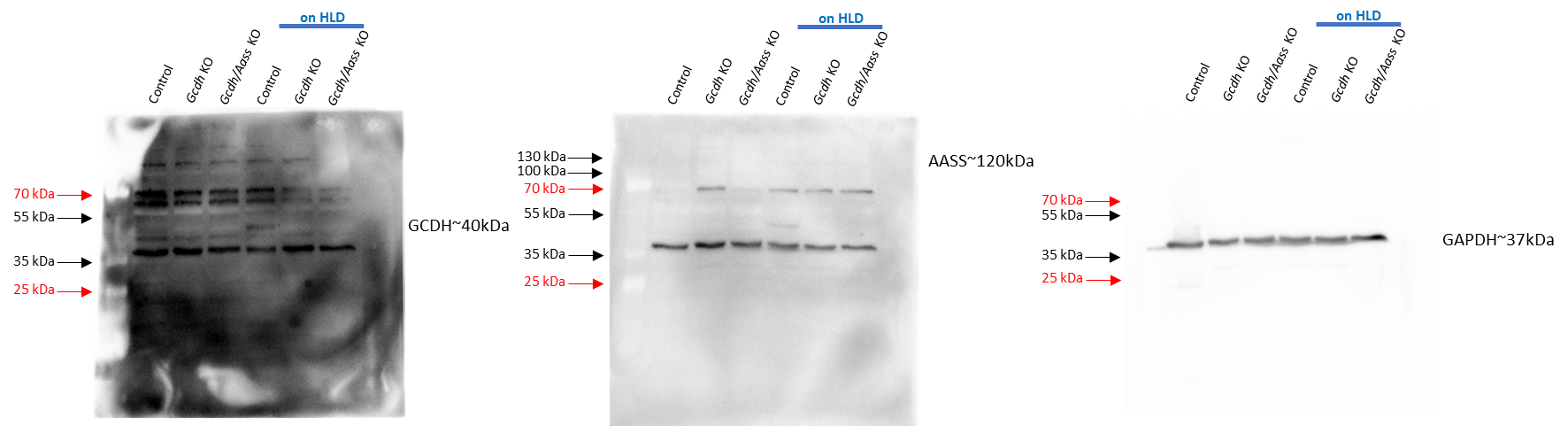
**Brain female**
3. **
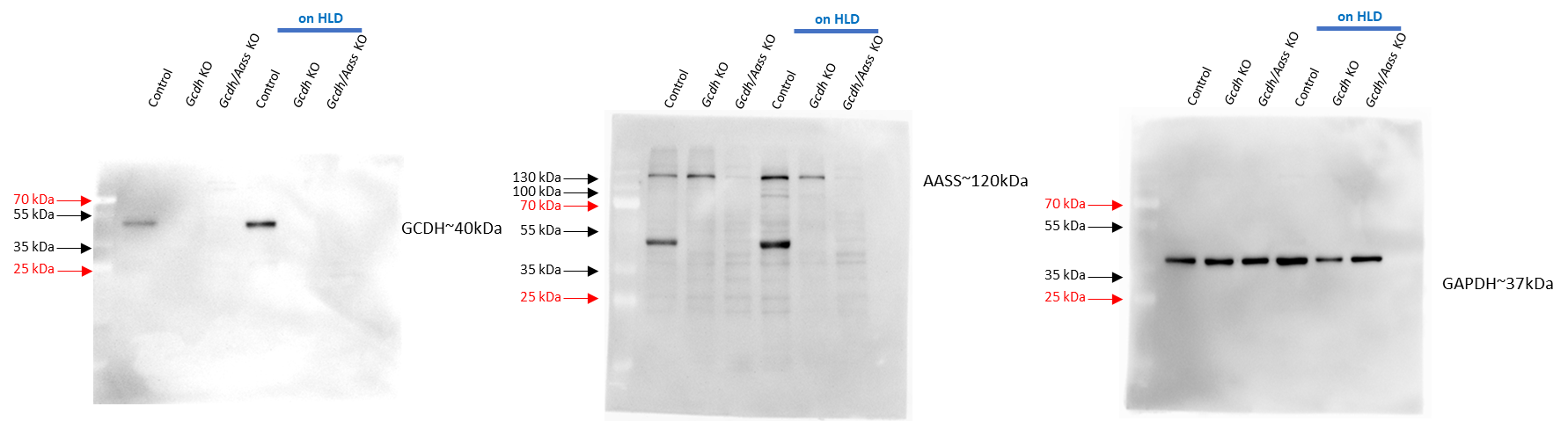
Liver male**
4. **Liver female**


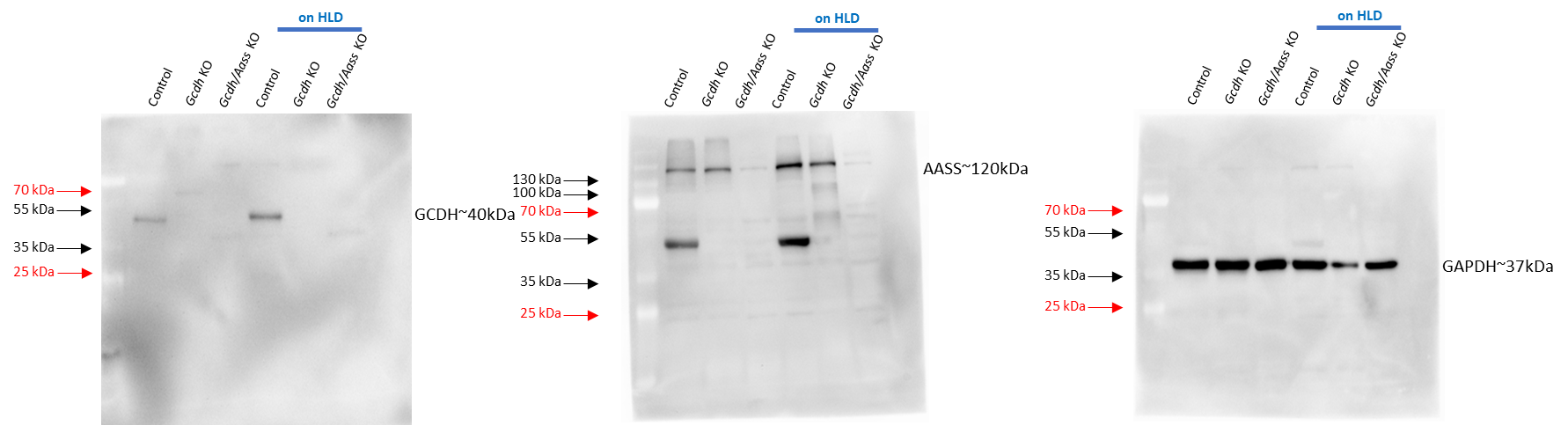


1. **
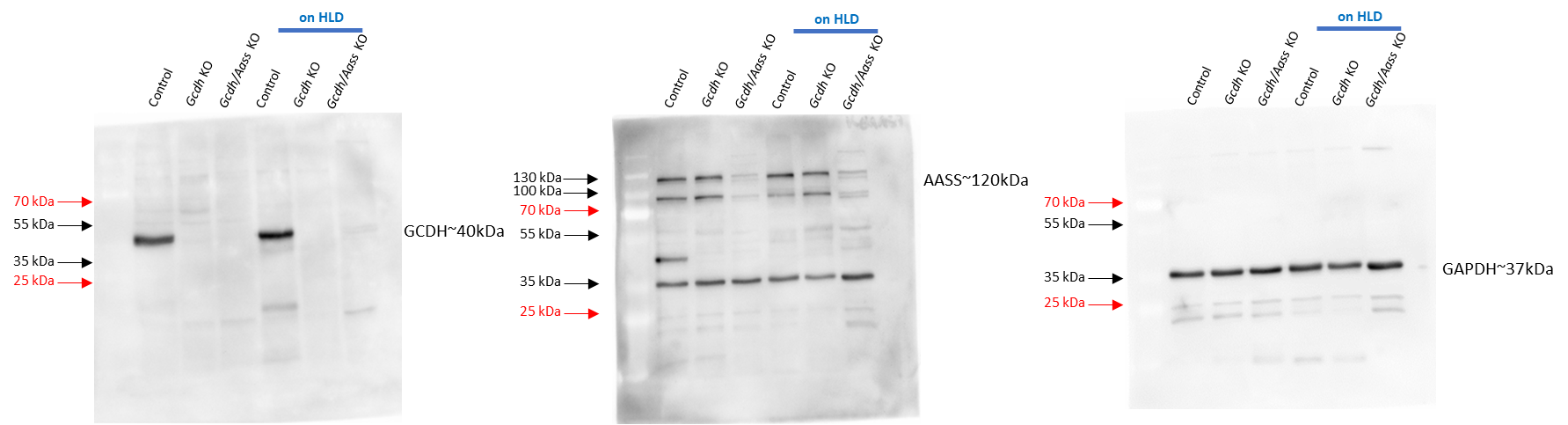
Kidney male**
2. **
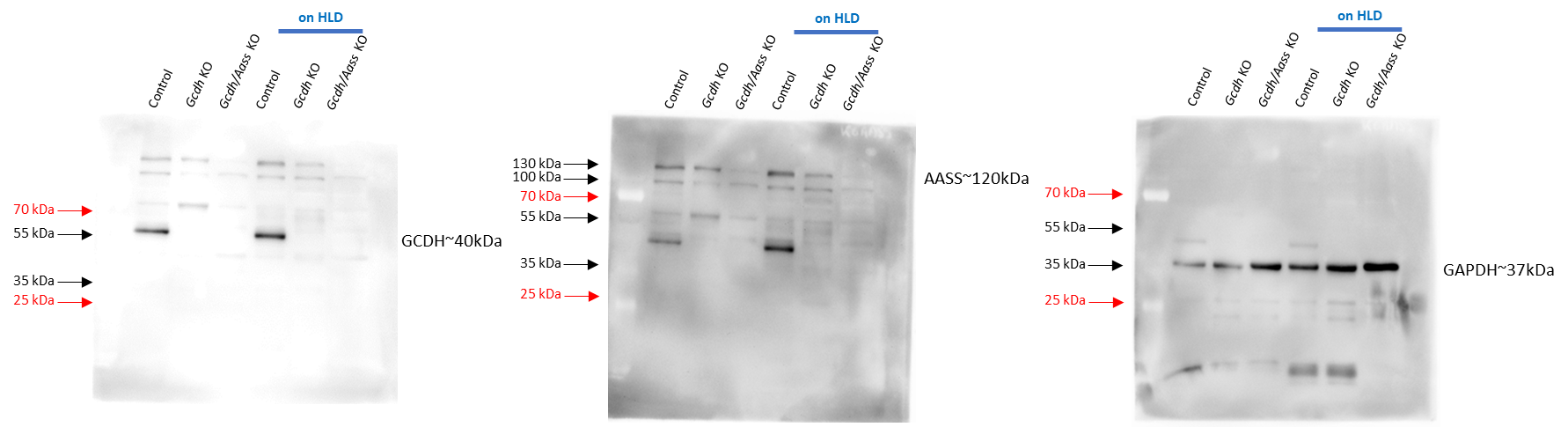
Kidney female**
3.
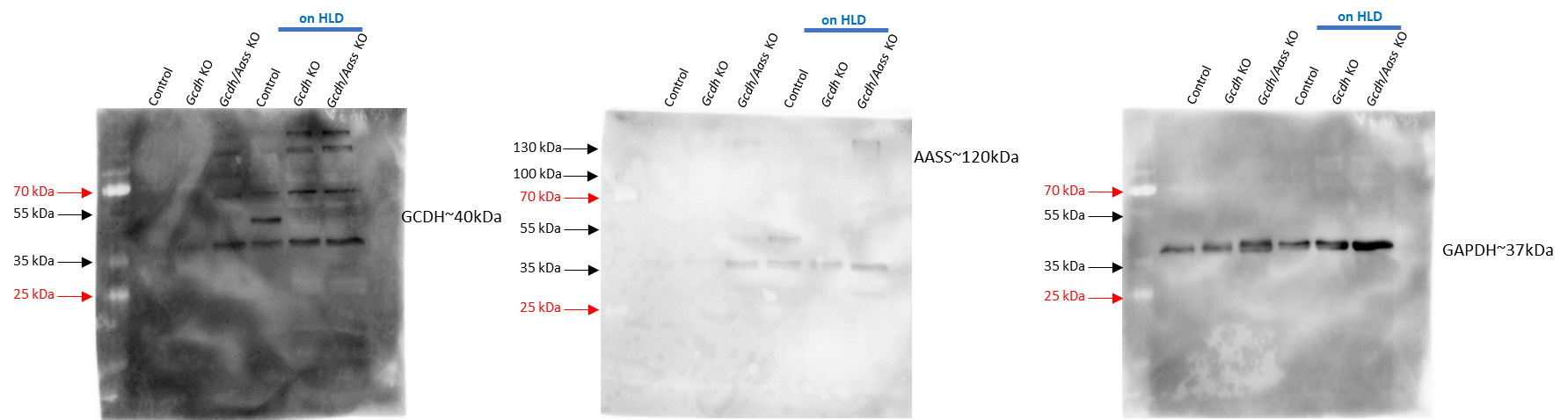
**Heart male**
4. **
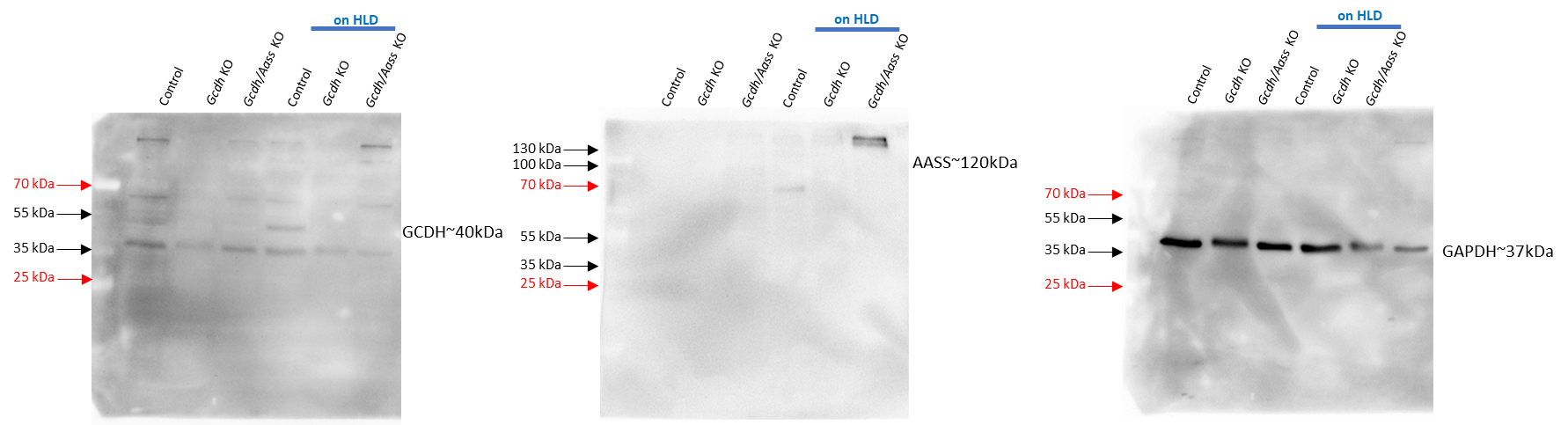
Heart female**
